# Description of *Ficus carica* L. Italian Cultivars—I: Machine Learning Based Analysis of Leaf Morphological Traits

**DOI:** 10.3390/plants14030333

**Published:** 2025-01-23

**Authors:** Cristiana Giordano, Lorenzo Arcidiaco, Margherita Rodolfi, Tommaso Ganino, Deborah Beghè, Raffaella Petruccelli

**Affiliations:** 1 Insitute of BioEconomy, CNR, via Madonna del Piano 10, Sesto Fiorentino, 50019 Firenze, Italy; cristiana.giordano@cnr.it (C.G.); tommaso.ganino@unipr.it (T.G.); raffaellaantonietta.petruccelli@cnr.it (R.P.); 2 Food and Drug Department, University of Parma, Parco Area delle Scienze, 27/a, 43124 Parma, Italy; margherita.rodolfi@unipr.it; 3 Economics and Management Department, University of Parma, Via J.F. Kennedy 6, 43125 Parma, Italy; deborah.beghe@unipr.it

**Keywords:** fig tree, trichome analysis, morphometric descriptors, pca, random forest, likelihood ratio test

## Abstract

Common fig, or simply fig (*Ficus carica* L.), is one of the most ancient species originated and domesticated in the Mediterranean basin. The Italian fig germplasm consists of a large number of cultivars, more than 300. This number is approximate; there are many genotypes that are still poorly known and studied that may possess interesting agronomic traits, especially in terms of response to climate change. Therefore, it is extremely important to study and preserve agrobiodiversity, but more importantly to identify simple and rapid characterization methods to catalog “hidden” cultivated plants. In this study, geometric leaf morphometry was used to explore differences among fifteen Tuscan fig cultivars. In addition, the effectiveness of a machine learning (ML) algorithm to characterize cultivars was evaluated. The study analyzed two classes of cultivars, one of plants with predominantly three-lobed leaf shape, and one five-lobed. Thirty-three descriptors for the five-lobed and twenty-three for the three-lobed. Anova analysis showed statistically significant differences for all characters analyzed and allowed an initial characterization of the material. Then, Random Forest algorithm analysis was used to reduce the number of parameters to those most significant for classification. The results showed that machine learning-based techniques are a valid system for analyzing leaves of *F. carica* cultivars and interpreting significant differences in leaf parameters. Classification based on the Random Forest model allowed us to filter out the main descriptors that best differentiate cultivars from each other.

## 1. Introduction

The common fig (*Ficus carica* L.), described by Caroli Linnaei in *Species Plantarum* [1] as *Ficus “foliis plamatis*”, is a deciduous, perennial tree belonging to the family Moraceae. *F. carica* is an excellent example of a fruit tree from the Mediterranean basin, where natural populations were present before domestication. It appears that in the Mediterranean basin area the cultivated fig was domesticated about 7000 years ago [2,3] from wild plants belonging to the genus *Ficus*. Zohary et al. [4] consider the fig, associated with the olive tree, grapevine, date palm, and pomegranate, one of the most important traditional fruits of Old-World agriculture that gave rise to early horticultural practices. The fig tree arrives in southern Italy (Magna Graecia, Sardinia) probably brought by the Greeks or Phoenicians between the 3rd and 8th centuries, as evidenced by archaeological findings [5,6], and then spread to the rest of the peninsula [7]. It has been harvested or cultivated for its edible fruit, consumed both as fresh and dried figs. Today, this perennial tree is widespread in all regions of the country; specifically, in the Piemonte region it is casual allochthonous, in Trentino and Valle d’Aosta it is naturalized allochthonous (Portale della Flora d’Italia). *Ficus carica* has great socioeconomic importance for many Mediterranean and Middle East countries which together produce 70–90% of the world’s supply of fig fruit. Worldwide, more than 1,000,000 tons per year are produced in an area of about 300,000 hectares [8]. Globally, about 30 percent of the fruit is consumed fresh in the domestic market, while 70 percent is consumed as dried figs [9]. Turkey is the country with the highest production (35% of global production) and it is also the biggest exporter of dried figs. Turkey exports 60–70% of the world’s dried fig production. Significant productions are also achieved in Egypt (19%), Algeria (13%), Morocco (12%), and Islamic Republic of Iran (7%). In Afghanistan, Tunisia, Albania, Brazil, Greece, China, India, USA and Japan are recent (or less recent) and important production areas. In Europe more than 100,000 tons per year are produced in an area of about 29,000 hectares; Spain contributes almost half of the entire European production, with about 40,000 tons on an area of more than 28,000 ha, followed by Italy, France and Portugal [8,10].

Italy is the second largest producer in Europe with 13,030 tons representing 12% of European production but only 1.3% of the world’s production (ISTAT 2023; http://dati.istat.it/ accessed on 20 January 2025). The largest contribution (80%) comes from the regions of southern Italy (Campania, Apulia, Calabria and Sicily) where cultivation in vegetable gardens and orchards is flanked by specialized crops, while more limited productions occur in areas of central-northern Italy (Tuscany, Liguria). All these regions differ, however, in the type of fruit produced. Calabria and Campania are characterized by the production of dried fruit, whereas in Apulia 90% of the production is marketed as fresh fruit [7]. In Tuscany and Liguria regions quality fruit production destined for niche markets is emerging. Italy has a rich varietal heritage. There are 24 cultivars of *Ficus carica* in the National Register of Varieties [11], but according to the Ligs4fun website, the main fig cultivars found in Italy are about 240 conserved both in private collections (Pomona Gardens in Apulia, or at production nurseries) and in national organizations, like CREA-MiPAAF (Research Centers for Horticulture, Fruit Growing and Citrus Farming in Rome; and Caserta) or University of Bari (Research Center for Experimentation and Training in Agriculture Basile Caramia Locorotondo; Bari, Italy) [7].

**Figure 1:**
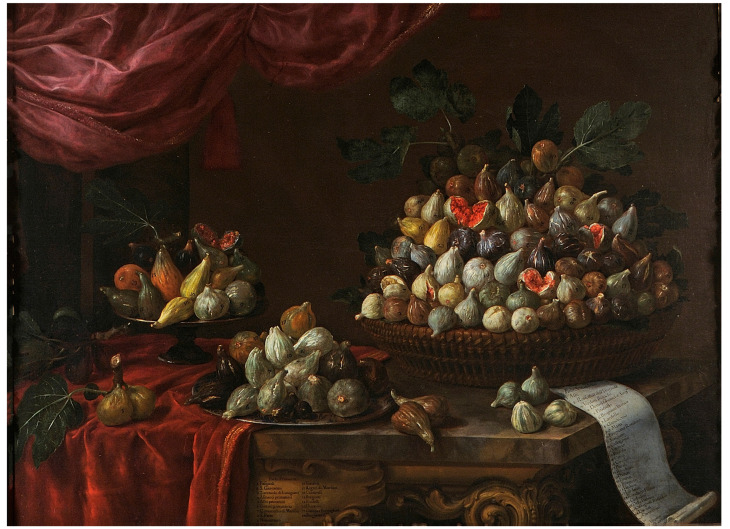
Bimbi Bartolomeo, 1696, Figs, oil on canvas. Prato, Poggio a Caiano, Museo della Natura Morta. Credit: “By concession of the Ministero della Cultura—Gabinetto Fotografico delle Gallerie degli Uffizi”. Inv. Oggetti d’Arte Castello (1911) n. 614.

Fig has been part of the Tuscan tradition for many centuries: the first evidence of its consumption dates back to the Etruscan period. In the sanctuary of the acropolis of Volterra, in a temple of the fifth century B.C., an abundance of achenes were found, probably related to deity cults [12]. The fig plant was painted in 5th- and 4th-century B.C. tombs in lower Etruria (Tarquinia) [13] along with myrtle, laurel, and palms; the mineralized fruit was found in the excavation of a small Roman farm in Cinigiano, southern Tuscany [14] dated from the late 2nd century B.C. to the late 1st century B.C. At the archaeological site of the Etruscan and Roman port of the city of Pisa, fig wood was used to build the C-ship frame, which dates to the 1st and 2nd centuries A.D [15]. Abundant fig achenes have been found in archaeological excavations in central Florence dating from late Roman to medieval times: the extreme abundance of achenes found in several levels of excavation suggests that the fruit was not only consumed in the period of production but also dried [16]. The earliest written records of its cultivation date back to the 1200s and are of fiscal and cadastral origin. In 15th-century writings, the names of the Verdino, Brogiotto Bianco, and Nero cultivars are given in “*contado fiorentino*” (which stands for the countryside around Florence), showing that the crop was well established in the area [17,18]. Although fig cultivation was of limited importance, Tuscany was enriched with cultivars thanks to the zeal of the Florentines and especially the passion of the Medici grand dukes, who loved to collect new “breeds”. The court botanist Pier Antonio Micheli (Pier Antonio Micheli 1679–1737) described the cultivars that arrived on the Medici table, and that grew in the gardens of the Medici villas and Bartolomeo Bimbi (Bartolomeo Bimbi 1648–1729) immortalized them in paintings posted in the rooms of the villas, providing a “splendid” catalog depicting 43 cultivars of figs (Figure 1). Figs of the Dottato cultivar, stored in boxes filled with millet, were usually shipped from Tuscany to Paris and Vienna, while dried figs were prized mainly in Lombardy [19]. The richness of the varietal heritage present in Tuscany reached 100 cultivars of figs at the beginning of the 19th century [20]. In the late 19th and early 20th centuries, fig cultivation, almost exclusively consociated, took place in rural areas where easily storable fruits such as dried figs were preferred [18]. The decline of sharecropping was accompanied by a gradual contraction of area and production, and by the end of the 1960s, the product was mainly supplied by plants scattered throughout the territory (about 80%) and limited by secondary crops (14%). Currently, annual production is only 97 tons, and the cultivated area is about 28 hectares (ISTAT 2023; http://dati.istat.it/ accessed on 20 January 2025). Although it is a minor crop in the Italian horticultural landscape, the fig has been receiving increasing attention in recent years both for the nutritional characteristics of the fruit and for its ability to adapt to climate change, with torrid climates and low water availability. For this reason, several studies and projects have been initiated for the conservation, enhancement and characterization of native germplasm. In relation to the latter, genetic and morphological characterization studies have been undertaken for cultivar description [21,22].

For a long time, characterization was conducted with the analysis of tree, leaf and fruit morphological characters. The classification of fig cvs presents problems related to the richness of the entities in the area, which are not always adequately identified. In fact, there is often, for historical or geographical reasons, a high degree of approximation, inaccuracy and inconsistency in the identifications of locally cultivated accessions. Individuals have multiple scientific names, or only common names, or present cases of synonymy or homonymy.

Although the reliability of morphological characterization is a matter of debate, since many morphological and agronomic traits depend significantly on environmental conditions, tree age, and stage of plant development, this approach continues to be the key initial step in describing and classifying fig germplasm. Consequently, the selection of highly distinctive variables is crucial to optimize resources and enable an effective morphological characterization. Morphometric traits are useful for cultivar identification because they are simple to collect, applicable in different agricultural settings, and easily understood by different stakeholders. In particular, leaf morphology is considered a stable parameter and therefore a valid representative method of cultivar identification [23].

Giraldo et al. [22] included leaf morphology (number of lobes and shape of lamina) among the main discriminating characters. Some authors have recently reintroduced leaf character analysis as a valid method of varietal identification from a geometric morphometric point of view; since it is a stable character, statistical analysis is more effective and powerful [23,24].

Different morphological and geometric traits have been used to analyze the diversity of fig cultivars. Recently, Ciarmiello et al. [25] conducted a study on the germplasm of Campania and Nuzzo et al. [24] on thirty local entities from Basilicata (South Italy) [25].

Machine learning (ML) methodologies are increasingly utilized for their ability to automate processes and improve accuracy in the analysis of morphological traits [26], as well as in the detection and classification of leaf diseases [27]. Among these methodologies, the Random Forest (RF) algorithm is particularly noteworthy due to its robust classification capabilities and its functionality in assessing variable importance (feature importance) [28,29,30]. This capability is essential for identifying the morphological parameters that most significantly contribute to the characterization and classification of cultivars. This comprehensive analysis aims to identify the morphological features that most effectively discriminate among the studied cultivars. These intrinsic characteristics led us to choose this classifier out of many. Furthermore, these techniques have demonstrated high accuracy in distinguishing cultivars based on leaf and fruit attributes. These methods leverage spectral properties, textural features, and morphological traits captured from images to achieve robust classification, often outperforming traditional approaches that rely on visual or manual inspection [31,32].

Fig represents a rich genetic diversity within Italian cultivars, with each variety exhibiting distinct morphological and biochemical traits. Identification of fig cultivars is critical for optimizing agricultural practices and ensuring quality control in both local and export markets. Despite traditional approaches to morphological classification, advancements in ML provide a transformative tool for analyzing the subtle differences in leaf morphological traits, offering higher accuracy and scalability [33].

This study investigates the potential of ML algorithms to classify Italian fig cultivars by analyzing morphological traits of leaves and to evaluate the feasibility and effectiveness of ML algorithm in association with geometric leaf morphometry to characterize fifteen fig cultivars. The model to be developed should allow the identification of the most discriminating morphological traits and the development of a classification model for fig cultivars.

## 2. Results and Discussion

### 2.1. Morphological Analysis

The present study was conducted in Carmignano (Prato, Tuscany), an important area for local traditional fig cultivars. Agromorphological and ethnobotanical bibliographic information was retrieved and used for subsequent morphological analysis [18]. The fifteen cultivars chosen for the present study are the most representative cultivars grown in the Tuscany region and have always been present on local historical farms. All the cultivars studied are maintained on a private farm. Table 1 shows the qualitative characters reported by IPGRI [34]. Both leaf margin character and central lobe shape had a prevalent shape in the fifteen cultivars: crenate margin and lanceolate central lobe shape. In contrast, the shape of leaf base was highly varied and showed a profile from cordate-calcarate-truncate in BN to simple calcarate in CO. All the fifteen cultivars showed a little lobe in central or lateral lobe, with marked differences among cultivars. The maximum value of little lobe in central lobe was 82.35% in PO, while the minimum value was 11.11% in AL. The same trend was observed for little lobe in lateral lobe, the values ranged from 100% in FI, to 5% in DO and BN (Table 1).

The number of lobes is a fundamental characteristic for the description of *Ficus carica* L. cultivars [22]. Those analyzed in this study had predominantly three-lobed leaves or five-lobed leaves (Table 1). Specifically, cultivars FI, SP, PO, VE, PA and BN were classified as “pure” three-lobed, while AL, CO, PN and PE were classified as “pure” five-lobed (Table 1). Five cultivars (BC, BB, GI, PB and DO) presented mixed leaf shape (three- and five-lobed). We included them in the class of three-lobed, since it was the predominant type. Considering this detail, different descriptors were used and therefore the two groups were analyzed separately (see Section 3.2).

Descriptive values for each quantitative trait are recorded in Table 2.

When examining the Standard Error (SE) and the Standard Deviation (SD), it is evident that these metrics are considerably higher for the WxH trait (9.8 and 165.2, respectively) and for BAC (3.1 and 52.7, respectively). This indicates a high level of inter-sample variability for these traits. In contrast, several traits exhibit low values of both SE and SD (Table 2). Considering the coefficient of variation (CV), the character with the greatest variability was L3y (CV 72.5%) followed by BAC (CV 44.6%) and I3y (36.6%). L2/L1 and β angle are the characters with least variability; 8.4% and 12.5%, respectively (Table 2).

For each group (three- and five-lobed), the results of ANOVA analysis are presented in Table 3 and Table 4. In the three-lobed cvs, considering leaf size, defined by the parameters lamina width (W) and lamina length (H), the values were as follows: 22.7 cm for BB and 16.9 cm for FI for the width; the largest value of H was recorded for DO (25.6 cm) and the least in FI (19.7 cm) (Table 3). Leaf area (WxH cm^2^) ranged from 580.6 cm^2^ (BB) to 338.9 cm^2^ (FI). According to IPGRI [35] the cvs BB and DO were included in the very large leaf area category (>550 cm^2^), while BN, GI, PA, PB and SP were classified in the large category (400–550 cm^2^); the remaining cvs were in the medium area category (250–400 cm^2^). Petiole length and width are morphological traits that can act as discriminators between cultivars, as reported in the literature and in *Ficus carica* L. species descriptors [22,34]. Our results showed statistically significant variations only for petiole length (PL), which ranged from 9.57 cm in BB to 6.95 cm in FI. All morphometric parameters analyzed showed a statistically significant difference between cultivars. The CLL/H ratio ranged from 0.60 in BB (which also recorded the highest CLL and H value) to 0.43 in VE (which recorded the smallest CLL value). The widest angle between basal lobes (BAC) was in SP (226.1°), while the one with the smallest angle was in PA (91.4°). Variation was also observed in the Zx and Zy values representing the coordinates of the point of maximum width of the central lobe; BB and BN showed the highest value of Zx (5.11 and 5.09 cm, respectively). The remaining morphometric parameters showed variability among the eleven cultivars; for example, the coordinates of the I2 point, representing the second sinus, ranged from 4.39 cm in BN to 2.83 cm in BC for I2x, while I2y was within a range of 11.4 cm in SP and 7.76 cm in BC. Wide statistically significant variability was observed in I2/L2 where the largest values were observed in SP and VE (0.76 and 0.77, respectively), while PB and BC showed the lowest values (0.52 and 0.58, respectively; Table 3).

Table 4 shows the results of ANOVA analysis for the five-lobed class. The cultivar CO showed the highest values for most of the characters analyzed. Among the morphological characters, W registered a range between 24.6 cm in CO and 18.5 cm in PN, while H was between and 30.5 cm in CO and 20.5 cm in PN. Leaf area (WxH cm^2^) ranged from 385.9 cm^2^ (PN) to 760.2 cm^2^ (CO). AL and CO can be classified into the category of very large (>550 cm^2^), PE in the large class (400–550 cm^2^), and PN in the medium class category (250–400 cm^2^), according to IPGRI descriptors. Among the morphometric parameters analyzed, the greatest variability was observed between CO and PN for all characters of I2, L2, I3, L3 and Z. The analysis of angles, petiole sinus (BAC) and angles between α and β lobes, is also interesting. For α and β, the highest values were observed in CO, 44.6° and 45.4°, respectively, and the lowest values are 37.9° and 37.5°, respectively, in PE. The BAC angle was reversed for CO, as it recorded the lowest value, 34.5°, while the highest value was observed in PN, 113.4° (Table 4).

Nuzzo et al. [24] found a relationship between petiole sinus angle (BAC) and leaf base shape. Our results showed a greater angle in cvs with a truncate base, as observed for the three-lobed cvs VE (BAC of 145.8°) and PO (BAC of 183.3°) or decurrent as in SP (BAC of 226.1°); with a lesser angle in cvs with a calcarate base shape, as in the five-lobed cvs CO (BAC of 34.5°) and AL with BAC of 91.5° (Table 1, Table 3 and Table 4).

Leaf morphological characters play a fundamental role in plant taxonomy and represent highly discriminating phenotypic variables in cultivar characterization. The morphometric characteristics of *Ficus* leaves have been investigated by a limited number of authors [36,37] who conducted studies on different species of the *Ficus* genus. Recently, Nuzzo et al. [24] in Italy and Abdelkader et al. [23] have used a multivariate morphometric approach to characterize autochthonous cultivars. The authors agree that leaf morphometric parameters can be simple and efficient systems to characterize even close cultivars.

#### 2.1.1. Selection of Features

Following a comprehensive evaluation of all morphological parameters, the ten most influential variables were identified based on their Gini importance scores, calculated using a Random Forest model applied to the two classes, three-lobed and five-lobed. This selection was achieved through a cross-analysis of the feature importance ranking plots.

**Figure 2:**
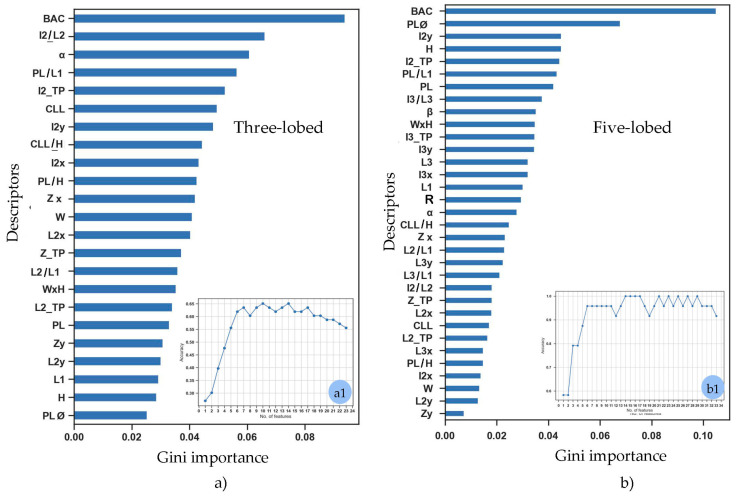
Comparative analysis of descriptor importance rankings, as measured by Gini importance, for distinguishing between three-lobed (**a**) and five-lobed (**b**) cultivars. The rankings are derived from a Random Forest classifier and highlight the most influential descriptors contributing to classification accuracy for each group. The embedded plots (**a1**,**b1**) show the performance curve in the ensemble increases. It demonstrates a saturation point where increasing the number of trees no longer significantly improves the model’s performance. These values are 7 for three-lobed cultivars and 6 for the five-lobed. Explanation of leaf descriptors is given in Sez. 3.2-Morphological Descriptors.

It was observed that for the three-lobed cultivars, seven variables were sufficient to achieve an accuracy of approximately 0.65, while for the five-lobed cultivars, six variables were adequate to reach an accuracy of 0.95 (Figure 2(a1,b1)). For three-lobed cultivars, descriptors like I2/L2, α, and PL/L1 occupy the second, third, and fourth positions, respectively in the top ranks, while for five-lobed cultivars, descriptors like PLØ, I2/TP, and H rank higher than in the three-lobed set, suggesting differences in the most discriminative traits for these groups. Many descriptors (e.g., BAC, I2y, PL/L1) appear in both plots with relatively high importance, indicating their general relevance across both cultivar groups. However, specific descriptors like α (three-lobed) and I3/L3 (five-lobed) highlight distinct morphological traits associated with the structural differences in the lobes. For the three-lobed class, the selected variables were BAC, I2/L2, α, PL/L1, I2_TP, CLL, I2y, CLL/H, I2x, and PL/H; whereas for the five-lobed group, the variables included: BAC, PLØ, I2y, H, I2_TP, PL/L1, PL, I3/L3, β, and WxH.

The results highlighted the importance of BAC across both groups. It suggests a fundamental role in defining key structural differences between cultivars, possibly linked to lobe size, shape, or symmetry. Differences in rankings of descriptors such as α and H may reflect variations in the geometric or proportional characteristics specific to each cultivar type. The gradual decline in Gini importance for lower-ranked descriptors suggests that many features contribute minimally to classification. This indicates potential redundancy in the dataset, where only the top-ranked descriptors provide significant discriminatory power. Based on the rankings, descriptors like BAC, PLØ, and I2/TP in future analysis should be prioritized for developing efficient predictive models or classification systems, as they consistently appear in the top ranks. For three-lobed cultivars, additional focus on α and PL/L1 may improve model accuracy, whereas for five-lobed cultivars, emphasizing H and I3/L3 could yield better results.

**Figure 3:**
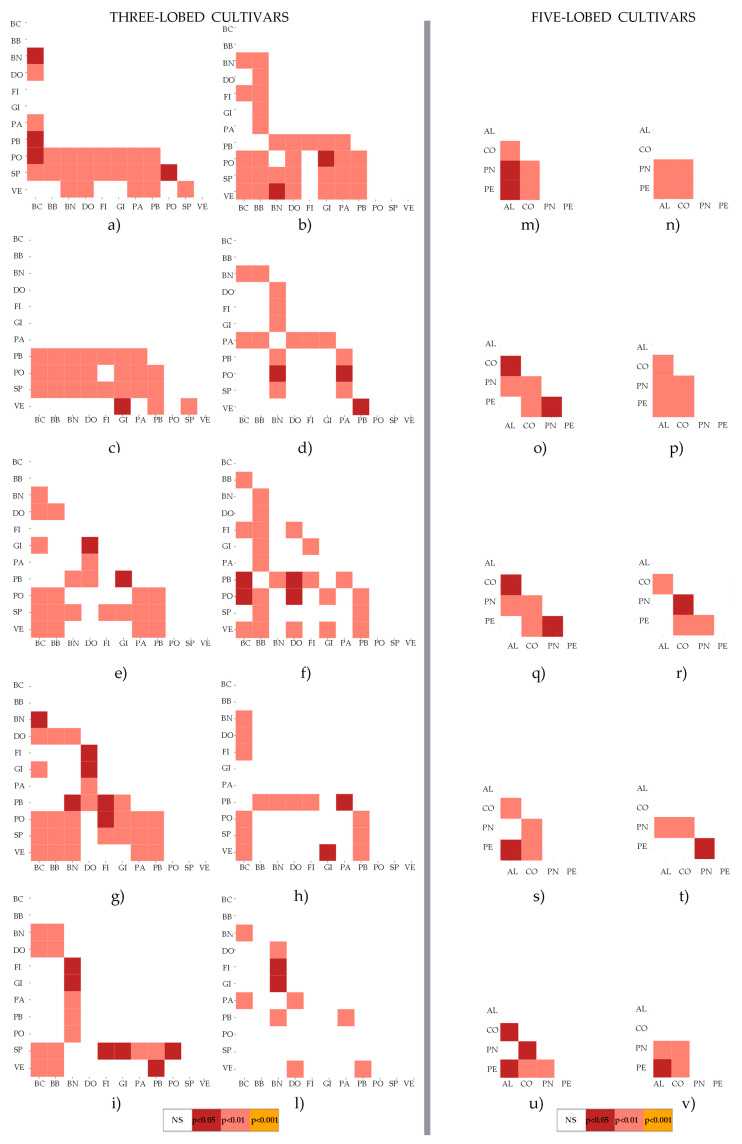
Heatmaps of *p*-values obtained from Tukey’s HSD test applied to the ten most significant descriptors of three-lobed (**a**–**i,l**) and five-lobed (**m**–**v**) *Ficus carica* cultivars. The selected descriptors for three-lobed cultivars are as follows: (**a**) BAC; (**b**) I2/L2; (**c**) α; (**d**) PL/L1; (**e**) I2_TP; (**f**) CLL; (**g**) I2y; (**h**) CLL/H; (**i**) I2x; (**l**) PL/H. The selected descriptors for five-lobed cultivars include: (**m**) BAC; (**n**) PLØ; (**o**) I2y; (**p**) H; (**q**) I2_TP; (**r**) PL/L1; (**s**) PL; (**t**) I3/L3; (**u**) β; (**v**) WxH. Significance levels are reported in legend by different colors. Codes for cvs are reported in Table 1; explanation of leaf descriptors is given in Sez. 3.2-Morphological Descriptors.

Figure 3 shows the Heatmap diagrams of the ten most important descriptors according to the RF algorithm, for both the three-lobed group (Figure 3a–i,l) and the five-lobed group (Figure 3m–v). In the three-lobed group, the cultivars PO, SP, and VE exhibit a higher frequency of significant differences at the 0.01 confidence level for the descriptors BAC, I2/L2, α, I2_TP, and I2y (Figure 3a–c,e,g). Conversely, for the descriptors PL/L1, CLL/H, and PL/H, the number of significant differences is lower across all confidence levels (Figure 3d,h,l). For the five-lobed group, the Heatmaps in Figure 3 reveal that the most significant differences occur between the PN and CO cultivars for all analyzed descriptors (*p* > 0.005; *p* > 0.01). The same cultivars, PN and CO, also demonstrate marked differences compared to the cultivars PE and AL. Specifically, five descriptors differentiate PN from PE (I2y, I2_TP, PL/L1, I3/L3, and β; Figure 3o,q,r,t,u), whereas eight descriptors distinguish PN from AL (BAC, PLØ, I2y, H, I2_TP, I3/L3, and WxH; Figure 3m–q,t,v). Regarding CO, statistically significant differences were observed for nine of the ten descriptors compared to PE (only I3/L3 was not significant, Figure 3t). However, no statistically significant differences were found for the descriptors PLØ, I3/L3, and WxH between CO and AL (Figure 3n,t,v). These findings are highly noteworthy as they clearly differentiate the four cultivars.

**Figure 4:**
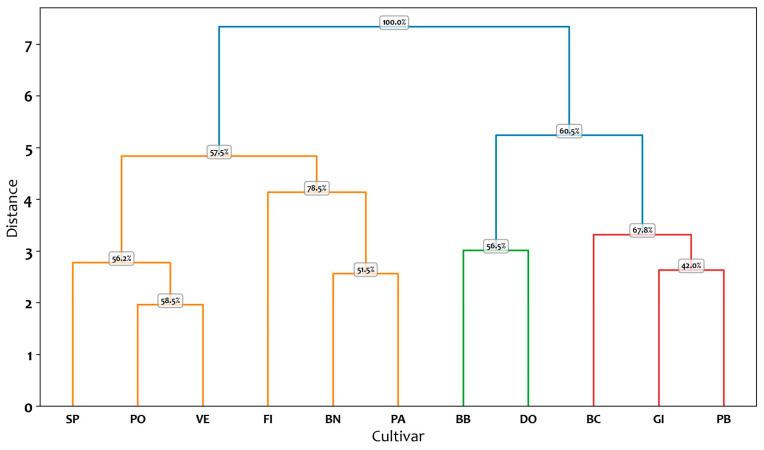
Cluster analysis of three-lobed cultivars based on ten descriptors selected by the Random Forest algorithm. The clustering method appears to group the entities based on their pairwise distances, as represented along the vertical axis (Distance). The horizontal axis lists the labels of the analyzed cultivars, and the branching structure indicates the hierarchical relationships among them. For each node in the dendrogram, the reported bootstrap values provide a measure of clustering reliability by assessing the stability of the clusters formed. The codes of the cultivars are reported in Table 1.

**Figure 5:**
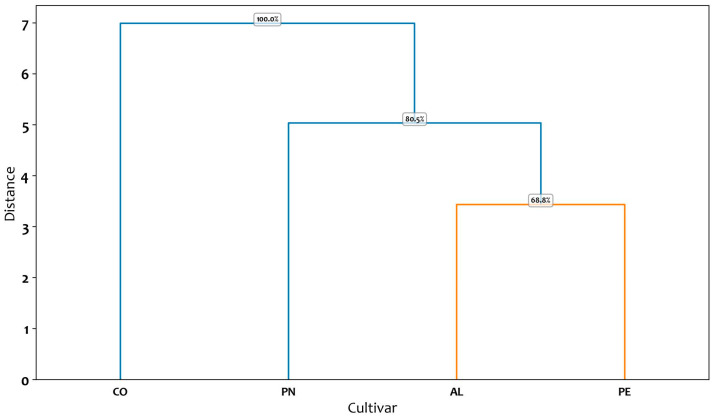
Cluster analysis of five-lobed cultivars based on ten descriptors selected by the Random Forest algorithm. The clustering method appears to group the entities based on their pairwise distances, as represented along the vertical axis (Distance). The horizontal axis lists the labels of the analyzed cultivars, and the branching structure indicates the hierarchical relationships among them. For each node in the dendrogram, the reported bootstrap values provide a measure of clustering reliability by assessing the stability of the clusters formed. The codes of the cultivars are reported in Table 1.

The tree-plots resulting from the cluster analysis of the ten descriptors selected by the RF algorithm, for the three-lobed and five-lobed class are shown in Figure 4 and Figure 5.

The cluster analysis of three-lobed cultivars, conducted using the Euclidean distance metric, revealed two main groups at a distance of 5.8 (Figure 4). Cluster 1 is further subdivided into two subgroups. The first subgroup includes cultivars PB, GI, and BC, with BC joining the cluster at a distance of 3.3. The common traits shared by BC, PB, and GI (or the shared characteristics within the first subgroup) are I2x and the ratios PL/L1, CLL/H, and PL/H. These three ratios are the unifying parameters for the entire first cluster, including the second subgroup comprising DO and BB. The second group is also divided into two subgroups. The first subgroup consists of PA and BN, which exhibit nine out of ten similar descriptors (with the exception of I2x). At a distance of 4, the cluster incorporates FI, which shares eight out of ten descriptors with PA and BN (including I2y, I2_TP, CLL, BAC and α angles, and the ratios I2/L2 and CLL/H). The second subgroup comprises VE and PO, which display similarity across all RF-selected traits, and at a distance of 3, SP joins the subgroup. SP shows statistically significant differences from VE and PO only for the traits BAC, and I2x.

Analyzing the bootstrap support, the hierarchical cluster analysis of Figure 4 partitions the examined cultivars into two principal clusters at a relatively large distance, as evidenced by the topmost branching node with a 100% bootstrap value. This high value indicates that the overarching split—separating the entire left group (SP, PO, VE, FI, BN, PA) from the right group (BB, DO, BC, GI, PB)—is consistently recovered in the majority of bootstrap resampling iterations, underscoring its robustness. Focusing on the left-hand site (in orange), several sub-branches reveal moderate-to-high support levels. For instance, the node separating SP from PO–VE has a bootstrap value of 56.2%, suggesting that SP frequently clusters on its own, while PO and VE typically group together (58.5%). At a higher level, the cluster containing FI, BN, and PA joins the PO–VE subtree at a node with 78.5% support. Notably, BN and PA share a branch with 51.5% support, reflecting some variability in how these two cultivars may be grouped under resampling. Collectively, these subclusters indicate that the finer-level splits among SP, PO, VE, FI, BN, and PA are fairly consistent, though not as robust as the primary division.

On the right side of the dendrogram, a node with a 60.5% bootstrap value unifies the entire cluster of BB, DO, BC, GI, and PB. Within this cluster, BB and DO form a green subgroup supported at 56.5%, indicating a moderate affinity between these two cultivars. In contrast, BC, GI, and PB form a red subgroup with slightly varying support levels: BC branches off at 67.8%, while GI and PB cluster at a lower bootstrap value of 42.0%, suggesting their grouping may be more prone to variation under resampling.

Overall, the upper-level partition—separating the left and right clusters at 100% bootstrap—is highly reliable, capturing the most pronounced differences among the cultivars. In contrast, lower-level groupings display moderate-to-lower support values (ranging roughly from 42% to 78.5%), implying that subtle distinctions among closely related cultivars can shift depending on the subset of data sampled. This pattern aligns with typical expectations in hierarchical clustering, where major divisions are frequently more robust, and finer-scale splits exhibit more variability in their bootstrap support.

The dendrogram obtained from the cluster analysis for the five-lobed class (Figure 5) highlights a clear distinction between the CO cultivar and the other three cultivars. The dendrogram partitions cultivars into two principal clusters at a relatively large distance, as demonstrated by the topmost branching node showing a 100% bootstrap value. This high value indicates that the overarching split—separating cultivar CO from the remaining three (PN, AL, PE)—is consistently retrieved in virtually all bootstrap resampling iterations, highlighting its robustness.

Focusing next on the separation of PN from the subcluster that includes AL and PE, the node has a bootstrap value of 80.5%. Although this value demonstrates that PN tends to branch off on its own in most resampling runs, it is not as absolute as the primary division. Finally, the grouping of AL and PE shows a 68.8% bootstrap value, suggesting that while these two cultivars frequently cluster together, their pairing is less stable than the top-level splits—likely reflecting subtler differences in the traits measured.

Overall, these results indicate that the highest-level partition in the dataset—between CO and the other cultivars—is exceptionally reliable (100%), whereas lower-level subdivisions (e.g., separating PN from AL and PE, and then grouping AL with PE) exhibit moderate support.

This pattern aligns with the expectation that major clusters, capturing the most pronounced differences, remain robust across bootstrap resamples, while finer-scale splits can fluctuate due to subtler trait variations.

**Figure 6:**
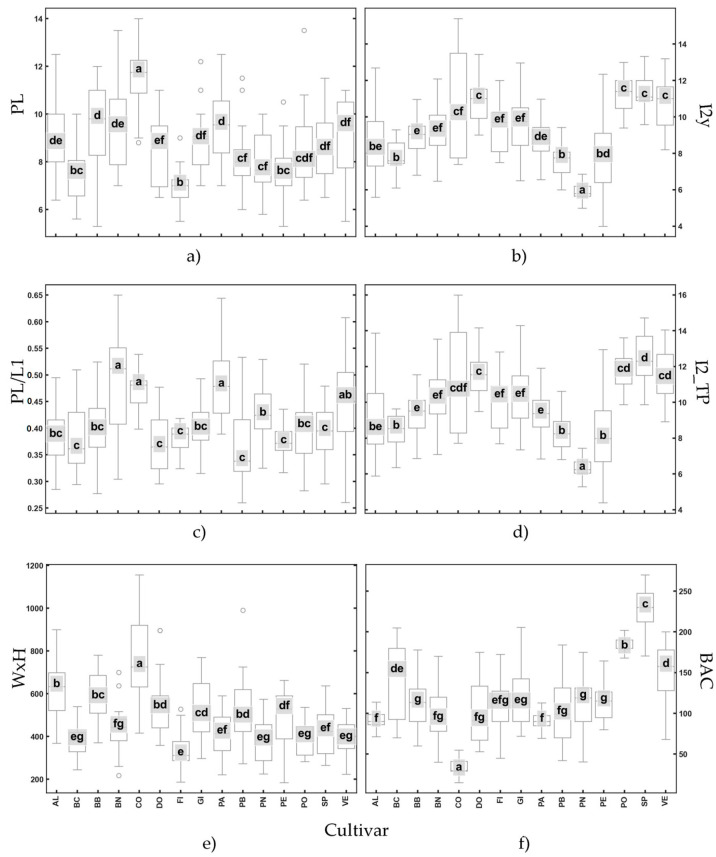
Box plots of the six most significant variables: (**a**) PL; (**b**) I2y; (**c**) PL/L1; (**d**) I2_TP; (**e**) WxH; (**f**) BAC. The letters a–g, represent the significantly difference at (*p* > 0.005). Codes for cvs are reported in Table 1; explanation of leaf descriptors is given in Sez. 3.2-Morphological Descriptors.

Figure 6 illustrates the distribution of the six variables selected across multiple cultivars using boxplots. Each panel represents a distinct trait, with individual boxplots corresponding to different cultivars. The letters above the boxplots indicate statistically significant groupings based on a post hoc analysis, highlighting differences between cultivars. CO is confirmed as the cultivar with the highest absolute values for almost all parameters.

- Panel a: variable PL shows considerable variability among cultivars, with statistically significant differences evident across groups (e.g., cultivar CO differs significantly from cultivars BC and FI).
- Panel b: variable I2y show statistically significant differences for all cultivars except for SP, PO and VE, which show similar values.
- Panel c: variable PL/L1 displays a narrower range, indicating lower variability, with several cultivars sharing overlapping groups (e.g., BC, DO, PB, PE, and SP).
- Panel d: trait I2_TP reveals intermediate variability, with notable outliers in certain cultivars, such as CO and PE.
- Panel e: variable WxH demonstrates the greatest variability, as reflected by the wider interquartile ranges and the presence of several outliers, such as CO.
- Panel f: trait BAC displays smaller interquartile ranges and a more consistent pattern, indicating less variability across cultivars. Significant differences evident for some cultivar (e.g., PO, SP, and VE compared with CO).

Overall, the figure highlights substantial inter-cultivar variability for several traits. PO, SP and VE exhibit similar values in almost all the descriptors (PL; I2Y; I2_TP, and WxH).

The Table 5 provides statistical analysis for comparisons among all the cultivars, divided into two sections: Pairwise comparison with the first three highest effect size values; Pairwise comparison with the last three lowest effect size values. The comparisons of the pair in the first group demonstrate strong statistical significance (very low *p*-values), large effect sizes, and high Bayesian factors (BF10), indicating robust evidence for the observed differences. The T-statistics are substantial (e.g., T = −25.5 for BAC: CO||SP) and *p*-values are exceedingly small (e.g., $p=1.59\times 10^{-25})$, showing highly significant results. The Confidence intervals (e.g., [−209.54, −178.75] for “BAC: CO||SP”) are narrow, indicating precise estimates of the effect. All comparisons show “Huge” or “Very large” effect sizes based on Cohen’s criteria (e.g., Effect Size = 11.90 for “BAC: CO||PO”) and the Bayesian Factor (BF10) values are massive (e.g., $BF10=1.833\times 10^{23})$, lending strong Bayesian support for the alternative hypotheses. Statistical power for all comparisons in this category is 1 (or very close), signifying an extremely high probability of detecting the true effect. Group pairs such as “CO||PO” and “PA||PO” in BAC, and “PN||SP” in I2y, exhibit the largest effects and highest Bayesian evidence. Despite the strong results, some comparisons (e.g., “PL_L1: DO||PA”) have slightly lower effect sizes (1.86) but still fall into the “Very large”. For this group the results reflect substantial, reliable differences among the compared groups, with consistent evidence from frequentist (*p*-values and effect sizes) and Bayesian (BF10) approaches. These findings are statistically and practically significant, warranting further exploration or consideration in decision-making. In contrast, the pair in the second group (Low Effect Size) indicates negligible or very small effects, with results lacking statistical significance. The T-statistics are close to zero (e.g., T = 0.1) and *p*-values are high (e.g., *p* = 0.92), showing no significant differences between groups. The Confidence Intervals (CI95%) include zero (e.g., [−21.5, 23.82]), reflecting uncertainty and a lack of evidence for meaningful differences. The effect sizes are classified as “Very small” or “Negligible” (e.g., 0.01–0.07), indicating minimal practical significance. BF10 values are close to 1 (e.g., BF10 = 0.31), suggesting weak evidence for either hypothesis. The power of tests in this category is approximately 0.05, indicating a low likelihood of detecting an effect even if it exists. Comparisons such as I2_TP: *BC||PE* and PL: *BB||BN* show extremely small effect sizes, reinforcing the conclusion of no practical significance. The pairwise results for this group indicate no noteworthy differences among groups, with negligible impact or practical utility.

**Figure 7:**
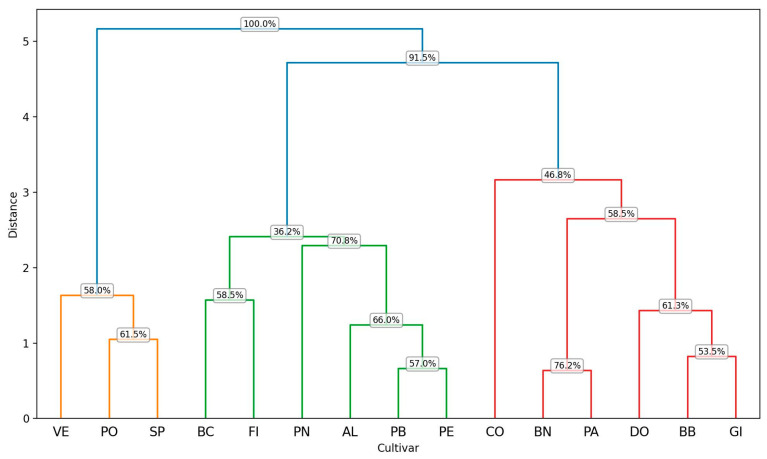
Result of a hierarchical cluster analysis based on the six most significant variables present in all the cultivars. The clustering method appears to group the entities based on their pairwise distances, as represented along the vertical axis (Distance). The horizontal axis lists the labels of the analyzed cultivars, and the branching structure indicates the hierarchical relationships among them. For each node in the dendrogram, the reported bootstrap values provide a measure of clustering reliability by assessing the stability of the clusters formed. The codes of the cultivars are reported in Table 1.

The hierarchical cluster analysis illustrated in Figure 7 partitions the examined cultivars into two principal clusters at a relatively high distance, evidenced by the topmost branching node displaying a 100% bootstrap value. This high value indicates that the fundamental split between these two overarching groups is consistently retrieved in the vast majority of bootstrap resampling iterations, underscoring its robustness.

Focusing first on the left-hand side of the dendrogram (in orange and green), cultivars VE, PO, SP form a distinct subcluster (orange) with moderate support values (e.g., 58% for the node separating VE and 61.5% for the node that groups PO and SP). Although these percentages suggest that the grouping appears in more than half of the bootstrap replicates, they are not as strong as the topmost division—reflecting some variability in the underlying traits for these samples. Meanwhile, the green subcluster (including BC, FI, PN, AL, PB, and PE) exhibits bootstrap values ranging from modest (36.2% at the node splitting off BC) to higher intermediate levels (66–70.8% for nodes connecting PN–AL and PB–PE). While these splits are fairly consistent overall, the moderate support values indicate that minor rearrangements can occur in the dendrogram under resampling, likely due to subtler morphological or numerical differences.

By contrast, the right-hand side of the dendrogram (in red) groups CO, BN, PA, DO, BB, GI together. Here, the node uniting CO with the rest shows a bootstrap value of 46.8%, revealing moderate-to-low support and suggesting greater potential for alternative placements of CO in different resampling runs. Within that cluster, the node unifying BN and PA has slightly stronger support (58.5%), whereas the splits involving DO, BB, and GI vary from moderate (61.3%) to relatively lower (53.5%). These intermediate or lower bootstrap values highlight finer-scale distinctions among these cultivars, which may shift under different bootstrap samples. Overall, the upper-level partition of the dataset—supported by a 100% bootstrap value—is highly reliable, confirming that the major separation among these cultivars is robust. Below this main split, different cluster groupings receive varying degrees of support, pointing to more nuanced trait similarities and differences that can cause slight rearrangements in the dendrogram. Consequently, while the top-level branching is consistently recovered, the lower-level subdivisions exhibit moderate to low bootstrap values, indicating a higher degree of uncertainty in the precise arrangement of some cultivars.

#### 2.1.2. Cultivar Classification by Random Forest

The Random Forest classifier was applied to all cultivars in the dataset using the six most significant common variables, yielding an overall accuracy of 0.49 (Table 6). shows the performance metrics of a Random Forest classifier in predicting various cultivars based on precision, recall, and F1-score. The results revealed that the RF model performs effectively for certain cultivars: FI and SP achieve a precision of 1, while PB and CO achieve precisions of 0.75 and 0.71, respectively. In contrast, PN and DO exhibit lower precision values, at 0.29 and 0.25, respectively. The recall metric (Table 6) indicates perfect classification (1.0) for CO and PO, whereas the lowest recall values are observed for PN, AL, at 0.33 and 0.20, respectively, and BN, GI both with 0.0. Analysis of the validation metrics using the F1-score highlights the highest values for SP, CO, and PO (0.89, 0.83, and 0.75, respectively), while the lowest scores are associated with AL, BN, and GI (0.29 for AL and 0.0 for BN and GI).

The following insights can be drawn:

- Class-wise Performance: Cultivars such as CO and SP show the best classification performance with high precision (0.71 and 1.00), recall (1.00), and F1-scores (0.83 and 0.89), indicating that the model accurately identifies these cultivars with few misclassifications. On the contrary, cultivars like BN and GI exhibit the poorest performance, with all metrics (precision, recall, and F1-score) at 0.00. This suggests that the classifier struggles entirely with these cultivars, likely due to class imbalance, lack of distinctive features, or other limitations in the dataset.
- Intermediate Performance: Cultivars such as PE, PO, and PB have moderate F1-scores ranging from 0.60 to 0.75. While the recall is strong for some, such as PO (0.60 precision, 1.00 recall), the precision-recall trade-off indicates room for improvement in minimizing false positives.
- Poor Recall for High Precision Cultivars: A notable case is FI, which shows perfect precision (1.00) but a recall of only 0.33, resulting in a moderate F1-score (0.50). This suggests that while the classifier is confident when it predicts FI, it fails to capture many actual instances, indicating underprediction for this class.
- Weighted Metrics: the weighted average precision, recall, and F1-score are 0.49, 0.49, and 0.47, respectively. These values reflect the overall performance across all cultivars, weighted by the number of instances in each class. The low values indicate that the classifier struggles to generalize well across multiple classes.
- Overall Accuracy: The overall accuracy of the classifier is 0.49, which is only slightly better than random chance in a binary context. This underscores the challenges the classifier faces in achieving consistent performance across cultivars.

**Figure 8:**
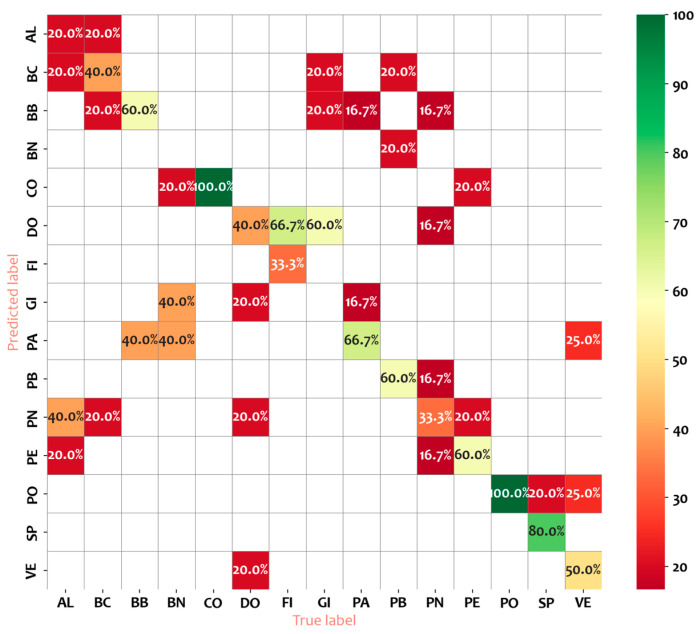
Confusing matrix derived from to RF classifier. The codes of the cultivars are reported in Table 1.

The results presented in Figure 8 showed that nine cultivars were classified with accuracy and specific values (≥50%), particularly CO, PO, and SP exhibited high classification accuracy (100%, 100%, and 80%, respectively). Notably, cultivar AL was classified with the lowest accuracy (20%) and is misclassified 40% of the time, primarily as PN. FI e GI were confused with DO with a rate of misclassification of 66.7%. Our results showed that the RF model is capable of recognizing differences in the six morphometric parameters analyzed and classifying cultivars. However, the results point out that there may be misclassified probably due to the plasticity of the leaf, which affects the results of the model.

#### 2.1.3. PCA Analysis

Table 7 reports the first three components from the Principal Component Analysis (PCA) of the six most significant characters of all fifteen cvs studied, and it summarizes the results of a Principal Component Analysis (PCA), detailing the contributions of six selected traits to the first three principal components (PC1, PC2, and PC3), along with the associated eigenvalues and explained variance.

**Figure 9:**
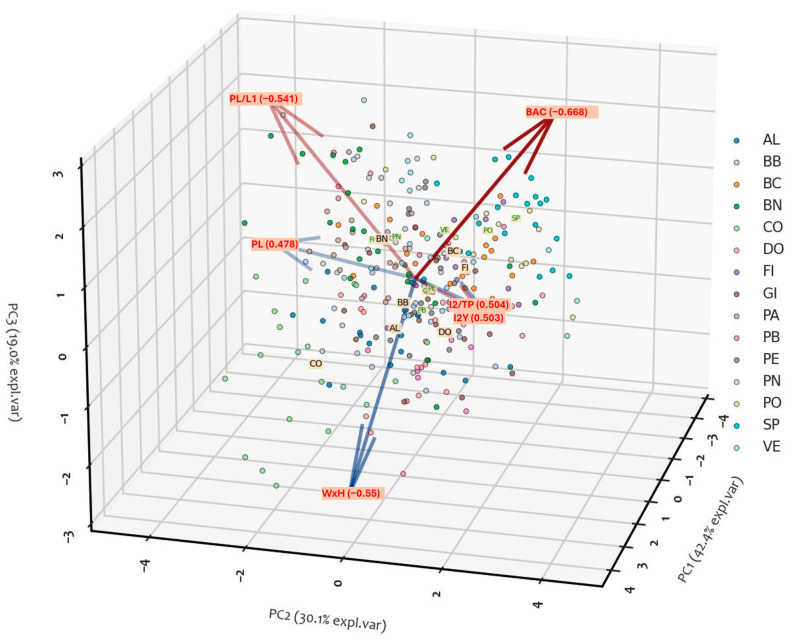
3D representation of PCA where the three dimensions are defined by the first three principal components. The descriptors used are: WxH; PL; I2_tp; I2y; PL/L1; BAC. Codes for cvs are reported in Table 1; explanation of leaf descriptors is given in Sez. 3.2-Morphological Descriptors.

The three components cumulatively explain 91.5% of the total variance. This indicates that most of the variability in the dataset can be captured by these three principal components. PC1 contributed the largest proportion of variability (42.4%) and is primarily influenced by I2y (loading: 0.51), I2_TP (loading: 0.50), and PL (loading: 0.48), with moderate contributions from WxH (0.45). PC2 explained 30.1% of the variance and is dominated by BAC (loading: 0.49) and I2_TP, I2y (both at 0.41), with a negative influence from PL_L1 (−0.51). PC3 accounted for 19% of the variance, with significant contributions from PL_L1 (0.60), BAC (0.51), and WxH (−0.55). The traits I2_TP and I2y exhibit strong positive contributions across PC1 and PC2, suggesting their importance in describing the major sources of variability. Conversely, PL_L1 and WxH contribute more prominently to PC3, indicating their relevance in capturing secondary patterns in the data. PCA reveals that the six traits are highly variable and are related to variability among cultivars. The analysis of the plot presented in Figure 9 revealed a distribution of samples with a weak degree of aggregation. Based on the length and direction of the feature vectors, it is evident that four features (BAC, PL-L1, PL, WxH) play a significant role in determining the principal components. However, these features also exhibit weak correlations among themselves. Conversely, the other two selected features (I2_TP and I2y) are strongly correlated (Figure 9).

### 2.2. Trichome Analysis

Trichomes are appendages that cover different organs of the plant; they originate from the cells of the epidermis and grow outward from the surface to a conspicuous size. Their location, shape, content and density play a key role in the plant’s defense against biotic and abiotic stresses. They provide a barrier in defense against pathogens [38], reflect solar radiation and filter harmful ultraviolet rays [39], absorb moisture and nutrients from the atmosphere [40,41] and reduce evapotranspiration by affecting temperature and photosynthetic rate [42,43]. Trichomes are valuable characters for taxonomic identification at different infra-generic levels and are usually used for classification purposes by many systematics [44,45]. Consequently, we assessed the presence of the trichomes of the upper and lower leaf surface of the studied cultivars. *Ficus carica* has glandular and non-glandular trichomes on the adaxial and abaxial surfaces of the leaf. The glandular ones are peltate; the non-glandular ones are unicellular, simple, spine-like of different sizes together with papillae-like lithocysts of the same shape but emerging from a shield-like base [46].

**Figure 10:**
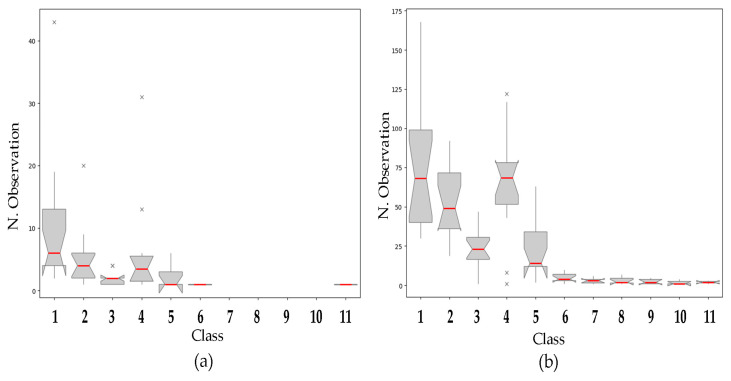
Distribution of trichomes by length classes. (**a**) upper epidermis. (**b**) lower epidermis. Class n. 1: 0.1–99 µm; class n. 2: 100–140 µm; class n. 3: 140.1–159 µm; class n. 4: 160–239 µm; class n. 5: 240–299 µm; class n. 6: 300–319 µm; class n. 7: 320–332 µm; class n. 8: 334–358 µm; class n. 9: 360–398 µm; class n. 10: 412–438 µm; class n. 11: 450–477 µm.

Both glandular and non-glandular trichomes were observed on the lower and upper pages. Most hairs on the epidermis were non-glandular, with a marked difference in size, characterized in this study in 11 length classes. Figure 10 shows the distributions of the different categories for the upper page (Figure 10a) and for the lower page (Figure 10b). Our results show that classes 1 (0.1–99 µm) to 5 (240–299 µm) are the most represented in both the upper and lower epidermis, with a clear predominance on the lower page (Figure 10a). Classes 6 to 11 are predominantly or exclusively observed on the lower leaf page (Figure 10a,b).

**Figure 11:**
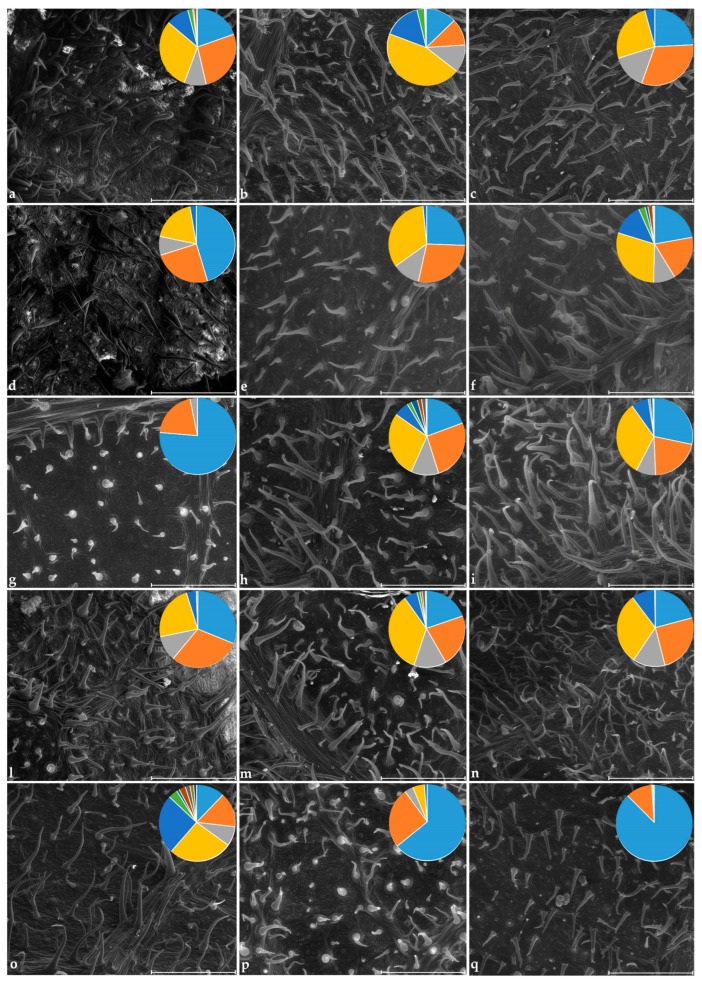
ESEM photographs. Trichomes on abaxial epidermis of *Ficus carica* and relative circle chart of the percentage distribution of hairs in 11 trichome length classes. The cultivars are (**a**): AL; (**b**): BC; (**c**): BB; (**d**): BN; (**e**): CO; (**f**): DO; (**g**): FI; (**h**): GI; (**i**): PA; (**l**): PB; (**m**): PN; (**n**): PE; (**o**): PO; (**p**): SP; (**q**): VE. Class colors: 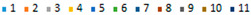. Class n. 1: 0.1–99 µm; class n. 2: 100–140 µm; class n. 3: 140.1–159 µm; class n. 4: 160–239 µm; class n. 5: 240–299 µm; class n. 6: 300–319 µm; class n. 7: 320–332 µm; class n. 8: 334–358 µm; class n. 9: 360–398 µm; class n. 10: 412–438 µm; class n. 11: 450–477 µm. The codes of the cultivars are reported in Table 1.

Table S1 shows the percentage distribution of the different classes in the studied cultivars for the upper and lower epidermis. The distribution in classes of the lower epidermis of the cultivars is also represented in the circle chart in Figure 11. The cultivars DO, PB, PO showed trichomes belonging to almost all 11 classes, while FI and VE registered hairs with sizes that fall only in classes 1 to 4 and therefore trichomes of shorter length.

**Figure 12:**
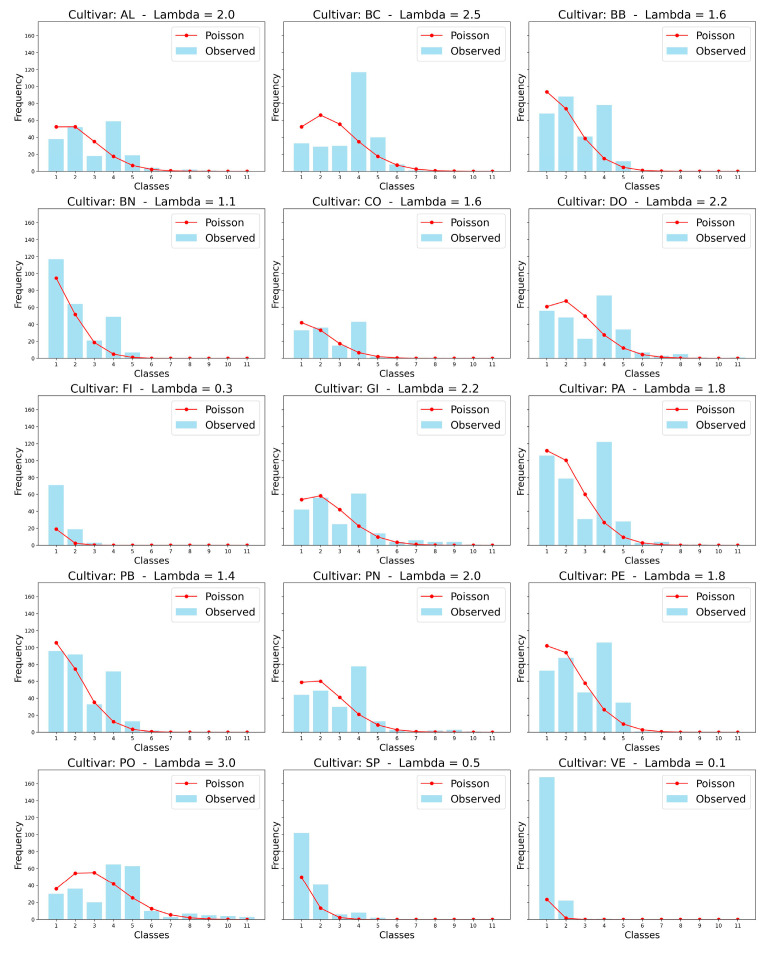
Distribution of observed frequencies of classes alongside modeled Poisson distributions for various cultivars, each characterized by a specific λ parameter.

The trichome frequency distributions by height class were reconstructed for the lower surface of the leaves, where their presence predominates. Subsequently, for each cultivar, the λ parameter of the Poisson distribution (Equation (2)) was derived from the observed distributions (see Section 3.4.1). Figure 12 presents the observed distributions alongside the corresponding Poisson distributions. Overall, the Poisson model demonstrates a strong alignment with the observed class frequencies across cultivars, indicating that these distributions can largely be modelled using a Poisson process. However, notable deviations occur in cultivars with extreme λ values, such as FI (λ = 0.3) and VE (λ = 0.1), where observed data diverge significantly from the predicted distributions. In contrast, higher λ values, such as PO (λ = 3.0), yield broader distributions encompassing a wider range of class frequencies, while lower λ values are associated with more concentrated distributions, with most observations confined to lower classes (Figure 12). These results suggest variability in the processes influencing class distributions among cultivars, likely driven by biological or environmental factors. The analysis underscores the utility of the Poisson model as a foundational tool for characterizing class frequency distributions, while also highlighting contexts where additional modelling may be required.

**Figure 13:**
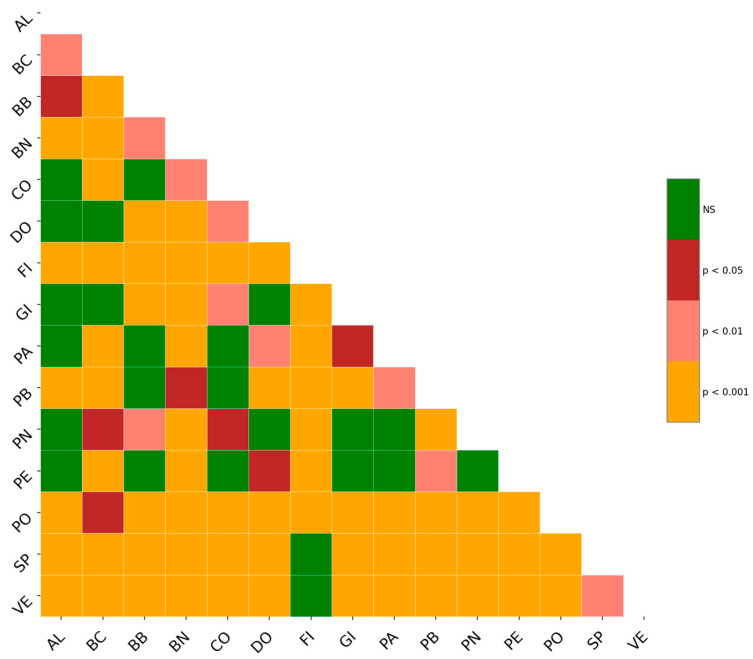
Statistical significance derived using Likelihood Ratio Test (LRT) in trichome distribution patterns. LRT statistical significance evaluated against a chi-squared distribution with two degrees of freedom. Significance levels are reported in legend by different colors. The codes of the cultivars are reported in Table 1.

Figure 13 reports the statistical significance of λ values evaluated using the Likelihood Ratio Test (LRT). The LRT statistic is assumed to follow a chi-squared (χ2) distribution with 2 degrees of freedom (see Section 3.4.1). The results indicate varying levels of significance across the cultivars, with certain pairs of Poisson distributions exhibiting highly significant differences in λ, suggesting distinct trichome density patterns. These findings highlight the utility of LRT for comparing Poisson-distributed data and underscore the heterogeneity in λ values among the cultivars, which may reflect underlying biological or environmental influences shaping trichome distributions. The most significant results (*p* < 0.001, in orange) are observed predominantly between cultivars with contrasting λ values, reflecting substantial differences in trichome density distributions. For example, cultivars such as PO, SP, VE and FI exhibit strong statistical distinctions from several others (Figure 13). This suggests significant variability in the underlying biological or environmental factors influencing trichome density. Conversely, non-significant comparisons (green, NS) are scarce and mainly occur among cultivars with similar λ values, such as PA and PE (λ = 1.8), indicating closely related trichome distribution patterns. These findings underscore the effectiveness of LRT-tests in identifying cultivars with significantly divergent traits and suggest that some pairs may represent distinct phenotypic or genetic groups.

Table 8 shows the density of hairs of the superior and inferior leaf page; high variability can be observed in both surfaces. For the upper page, the highest density value was observed in AL (26.3 trichomes mm^−2^) and the lowest in VE (1.02 trichomes mm^−2^). For the lower page, PA and PE had the highest density value (93.8 and 87.5 trichomes mm^−2^ respectively), while VE showed the lowest value with 48.2 trichomes mm^−2^.

**Figure 14:**
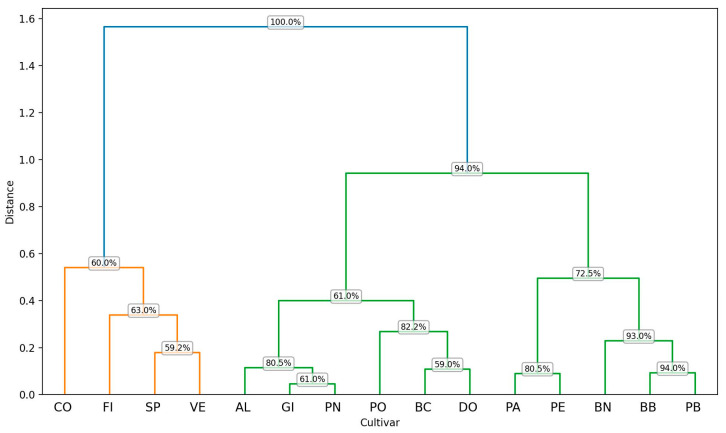
Dendrogram of the studied cultivars, derived using Lambda value of the respective Poisson distribution and density values. For each node in the dendrogram, the reported bootstrap values provide a measure of clustering reliability by assessing the stability of the clusters formed. The codes of the cultivars are reported in Table 1.

Based on the calculated λ parameters and the average trichome density, a hierarchical cluster analysis was performed using Euclidean distance (Figure 14). The results showed that, with a relative distance of 0.6, the fifteen cultivars clustered into three main groups. Cluster 1 clustered four cultivars (CO, FI, SP, and VE) that can be identified by a low density of trichomes on the lower page and the presence of trichomes that are concentrated in a limited number of classes in both epidermises. Cluster 2 aggregated six cultivars into two sub-clusters. The cultivars GI and PN, present in the first sub-cluster, were positioned very close to each other. In the second sub-cluster PO cv was closely related with BC and DO. The cultivars in cluster 2 were typified by the larger size of the hairs and an average density in the lower page. Cluster 3 encompassed five cultivars; at a dissimilarity level of 0.4–0.5, it divides into two sub-clusters. PE and PA, BB and PB contained in the first and second sub-clusters, respectively, are close to each other for the characteristics of trichomes (Figure 14). Focusing on the left cluster (containing CO, FI, SP, and VE), the node branching FI and SP from CO has a bootstrap value of 60%. While this suggests that the subcluster is observed in more than half of the bootstrap replicates, it is less stable than the main split, potentially reflecting moderate variability in the underlying morphological or numerical traits for these samples. Similarly, the split isolating VE (59.2%) exhibits a comparable level of consistency, indicating that, although VE typically segregates from CO, FI, and SP, the exact point at which it branches off can vary under different bootstrap samples.

In contrast, the right portion of the dendrogram (including AL, GI, PN, PO, BC, DO, PA, PE, BN, BB, PB) shows varying degrees of stability, with bootstrap values ranging from moderate (61% at the node separating AL, GI, and PN from PO, BC, DO, PA, PE, BN, BB, PB) to relatively high (over 90% for the splits involving BN, BB, and PB). Nodes with higher bootstrap values, such as 93% or 94%, indicate groupings that are more consistently observed under resampling, suggesting stronger similarity among those cultivars. Conversely, nodes with more modest support (around 59–72%) highlight potential overlaps or subtler morphological differences that may shift slightly from one resample to another. Overall, these results indicate that the upper-level partition of the dataset is highly reliable, whereas lower-level subdivisions show varying degrees of stability. This pattern is consistent with the expectation that major clusters—capturing the most pronounced differences among samples—tend to remain robust, whereas finer-scale splits, which hinge on subtler distinctions, may fluctuate and thus receive lower bootstrap support.

## 3. Materials and Methods

### 3.1. Plant Material

The plant materials consisted of leaf samples from fifteen *Ficus carica* L. cultivars collected from the private farm “Petracchi” located in Carmignano (Prato, Tuscany, IT; 43°48′49″ N, 11°1′6″ E, 189 m. a. s. l.). Trees were cultivated under the same agro-environmental conditions and according to the standard procedures of organic cultivation. A total of 287 fully mature fig leaves were randomly collected (80 pentalobate and 207 trilobate, respectively) in the middle third of the shoot and measurements were conducted on the leaves belonging to the dominant form of the cultivar. The names of the cultivars and codifications are reported in Table 1.

### 3.2. Morphological Descriptors

**Figure 15:**
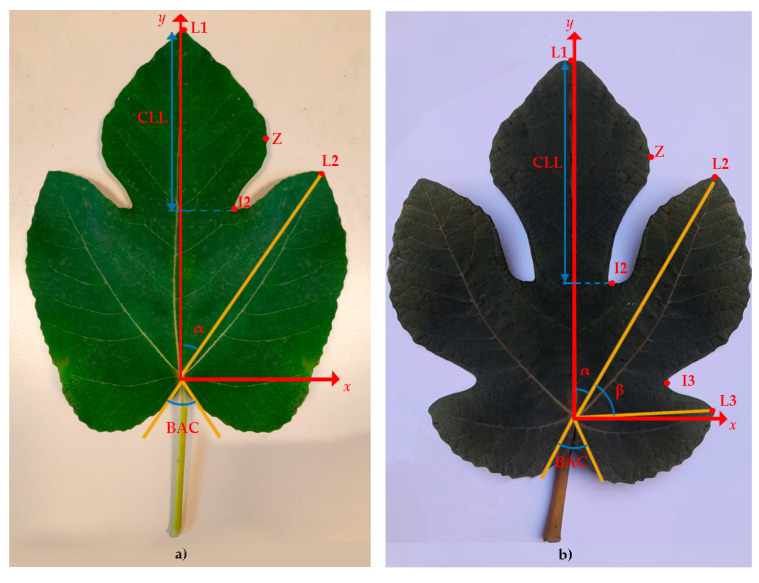
Morphometrical descriptors used in the study for (**a**) three-lobed leaf and (**b**) five-lobed leaf. The codes of leaf descriptors are reported in.

The morphological features were described using the methodology proposed by the International Plant Genetic Resource Institute [34] (Table 9). The qualitative descriptors analyzed were the number of lobes, leaf margin, shape of central lobe, presence and location of little lateral lobe, shape of leaf base. Quantitative leaf characters described were as follows: petiole length (PL; cm); petiole thickness (PLØ; cm); midrib length (L1; cm); leaf width (W; cm); leaf length (from the base of the blade to the tip of the central lobe; H; cm); leaf area (W*H; cm^2^); central lobe length (CLL; cm); leaf base angle (BAC; °); angles between main nerves (α = angle between L1 and L2; β = angle between L2 and L3; °). To these characters we added fifteen morphometric descriptors selected and measured only on the right side of each leaf, assuming that the leaves are symmetrical. The right half of the leaf was placed on a Cartesian plane with the origin at the point where the veins depart, and the y-axis superimposed on the central rib. For the following characters: Z (the maximum width of the central lobe, cm); L2 (apex of the second lobe; cm); L3 (apex of the third lobe; cm); I2 (sinus between lobe 2 and central lobe; cm) and I3 (sinus between L2 and L3; cm), x and y coordinates were measured. Moreover, the distance of the point from the center of the Cartesian plane was calculated using the Pythagorean Theorem (PT) applied to the x and y coordinates (Figure 15). In addition, the following ratios were calculated: I2/L2; L2/L1; I3/L3; PL/H; PL/L1; CLL/H (Table 9).

(1) $$R=\frac{\left(I2\_TP+I3\_TP\right)}{\left(L2\_TP+L3\_TP\right)}$$

### 3.3. Trichome Analysis

For trichome characterization the upper and lower surface of the leaf was analyzed using Fei Quanta 200 Environment Scanning Electron Microscope (ESEM), Fei Corporation, Eindhoven, The Netherlands, operating in low-vacuum mode (the chamber pressure was kept at 1 Torr), at 25 kV. Three pieces of tissue were cut from the central part of the leaf, left and right of the central vein, and analyzed without pre-treatment. The non-glandular trichomes were measured and divided into 11 frequency classes: class number 1 includes hairs measuring from 0.1 to 99 µm; class n. 2: 100–140 µm; class n. 3: 140.1–159 µm; class n. 4: 160–239 µm; class n. 5: 240–299 µm; class n. 6: 300–319 µm; class n. 7: 320–332 µm; class n. 8: 334–358 µm; class n. 9: 360–398 µm; class n. 10: 412–438 µm; class n. 11: 450–477 µm. Then, the hair density was calculated.

### 3.4. Statistical, PCA Analysis, and Random Forest Model

#### 3.4.1. Trichomes

Since each cultivar has a characteristic frequency distribution, the observed frequency distributions were analyzed, and the Lambda value of the respective Poisson distribution was derived for each cultivar (Equation (2)).

(2) $$P\left(x\right)=\frac{e^{-\lambda }\lambda^{x}}{x!}$$

The Lambda values thus obtained were compared by means of a Likelihood Ratio Test (LRT) to determine whether the observed differences between them were statistically significant. The Likelihood Ratio Test (LRT) is a statistical test of the goodness-of-fit between two models. A relatively more complex model is compared to a simpler model to see if it fits a particular dataset significantly better [8]. The LRT is based on evaluating the log-likelihood of two models: a null model, in which both cultivars are assumed to follow a common distribution, and an alternative model, in which each cultivar follows its own specific distribution. The test statistic is calculated as follows:

(3) $$LTR=2\times (\left(Log(likelihood\;of\;thealternative\;model\right)\;\;\;\;\;\;\;\;\;\;\;\;\;\;\;\;\;\;-\left(Log(likelihood\;of\;the\;null\;model\right))$$

Under the null hypothesis, the Likelihood Ratio Test (LRT) statistic is assumed to follow a chi-squared (*χ*^2^) distribution with degrees of freedom equal to the difference in the number of free parameters between the compared models. In this study, since the comparison involved two Poisson distributions differing by a single parameter (*λ*), the test statistic was evaluated with 2 degrees of freedom. The *p*-value for each pair of cultivars was computed to assess the statistical significance of differences between their respective distributions. This methodology enabled the identification of statistically significant differences in the Lambda parameter between the two cultivars, as reflected in their frequency distributions, across three significance levels (0.05, 0.01, 0.001).

Subsequently, the estimated Lambda and density values for each cultivar were utilized as input features for a cluster analysis. The analysis produced a dendrogram based on a Euclidean distance metric to determine linkage.

#### 3.4.2. Morphological Variables

A comprehensive data analysis of fifteen fig cultivars (Table 1) was conducted on the dataset containing thirty-two quantitative morphological variables (of which ten morphological and twenty-two morphometrical descriptors; Table 9). To mitigate the impact of potential outliers, the dataset underwent preprocessing, including data control, cleaning, and the application of a statistical transformation function to each morphological feature of single cultivar. For each cultivar and morphological trait was determined the 25^fh^ percentile (FQ), the 75^fh^ percentile (3Q). and the Inter Quartile Range (IQR) as IQR=3Q−1Q. Then the outliers values were selected and replaced with the Winsorization technique [47].

(4) $$values\leq \;\left(1Q-3.5\times IQR\right)\;were\;set=\left(1Q-1.5\times IQR\right)$$

(5) $$values\geq \;\left(3Q+1.5\times IQR\right)\;were\;set=\left(3Q+1.5\times IQR\right)$$

Unlike data trimming, which excludes extreme values, Winsorization replaces these values with defined percentiles, thereby preserving the dataset’s structure while reducing the influence of outlier.

Descriptive statistics were calculated for all morphological variables, including the mean, median, 1st and 3th quartile, Standard Error (SE; measures the uncertainty around the sample mean as an estimate of the population mean), Standard Deviation (SD; measures the variability within your data, indicating how spread out the individual data points are from the mean), Minimum, Maximum, and Coefficient of Variation (CV). These statistics were derived at three distinct levels: first, for whole dataset; second by grouping data based on the cultivar, and third, by grouping them into two classes defined by the number of leaf lobes: three-lobed and five-lobed.

To investigate whether significant differences existed in trait means across classes (three-lobed and five-lobed), an Analysis of Variance (ANOVA) was conducted using the F-statistic. Following the ANOVA, post hoc tests were performed to identify which specific group means differed while maintaining control over the overall Type I error rate. To quantitatively evaluate the morphometric differences between the cultivars, the significance of these differences was assessed using Tukey’s Honest Significant Difference (Tukey’s HSD) test [48] conducted at a 95% confidence level. Additionally, effect size (EF), Bayesian Factors (BF), and Confidence Intervals (CI) values were calculated to measure the magnitude of the observed effects relative to the standard deviation of the sample and the relative strength of the treatments. EF, BF, and CI values for each pairwise comparison were determined using Cohen’s method [49].

Prior to analysis, the dataset underwent standardization by centering the data (removing the mean) and scaling to unit variance. This preprocessing step ensures compatibility with many machine learning algorithms, which typically perform better when input data are normalized to have a mean of 0 and a standard deviation of 1.

#### 3.4.3. Random Forest Model

To identify the most influential morphological variables characterizing the cultivars, we applied the Random Forest (RF) classifier. Feature importance was assessed using the Mean Decrease in Impurity (MDI) metric, which quantifies impurity reduction based on the Gini index.

The original dataset was divided into train and test data by applying a proportion of 80/20 and the RF model was trained on the first subset, while the second subset was used exclusively for the validation phase. In order to ensure the reproducibility of the experiment, a randomstate = 10 was adopted. The significance of each feature was evaluated by examining the mean and standard deviation of impurity reductions. This process enabled us to identify the most influential variables, providing insights into the primary factors affecting our predictions. Separate analyses were conducted for cultivars classified as three-lobed and five-lobed, yielding ranked lists of feature importance for both groups. From these rankings, the top ten features were identified for each group, and the six features common to both were selected for further analysis. These six morphological features, representing key determinants of leaf morphology across all cultivars, were subsequently subjected to Tukey’s HSD test, independently of class distinctions.

To explore the clustering behavior of the cultivars, dendrograms were constructed for each class based on the selected six features, using Euclidean distance as the linkage criterion. In order to enhance the robustness and interpretability of the dendrograms, for each node we added the related bootstrap values. These values provide a measure of clustering reliability by assessing the stability of the clusters derived from resampling techniques where the data is repeatedly resampled, and the clustering process is reapplied. For each node in the dendrogram, a bootstrap value indicates the percentage of times a cluster was reproduced across all the resampled datasets. Bootstrap values are derived from resampling techniques where the data is repeatedly resampled, and the clustering process is reapplied. For each node in the dendrogram, a bootstrap value indicates the percentage of times a cluster was reproduced across all the resampled datasets. These values give an estimate of the confidence or stability of the clusters, much like how confidence intervals work in statistical analysis.

Additionally, Principal Component Analysis (PCA) was performed on six variables, for both three-lobed and five-lobed cultivars, facilitating a comprehensive understanding of the morphometric variations. To evaluate the contribution of each principal component to the total variance in the dataset, the explained variance for each component was analyzed by class. Subsequently, the contribution of the original variables to each component was examined to assess their respective influence. Finally, the relationships between the variables and the observed classes were visualized in a three-dimensional principal component space, with the axes representing the first three principal components.

The data analyst process also involved applying the RF model to the entire dataset and using the six descriptors common to the two classes.

In order to evaluate the performances of RF classifier we derived a Classification report calculated using the confusion matrices represented in a way to express how many of a classifier’s predictions were correct, and when incorrect, where the classifier got confused.

In the confusion matrices the rows represent the true labels, and the columns represent predicted labels. Values on the diagonal represent the number (or percent, in a normalized confusion matrix) of times where the predicted label matches the true label. Values in the other cells represent instances where the classifier mislabeled an observation; the column tells us what the classifier predicted, and the row tells us what the right label was.

To evaluate the model performance, we derived the following metrics:

Accuracy: represents the proportion of correct classified instances to the total:

(6) $$\mathrm{Accuracy}=\frac{\mathrm{Number}\;\mathrm{of}\;\mathrm{Correct}\;\mathrm{Predictions}}{\mathrm{Total}\;\mathrm{Predictions}}$$

A high accuracy suggests that the model made a large number of correct predictions in general. However, accuracy may not be a sufficient metric in cases of imbalanced classes (where some classes are much more frequent than others). In such cases, a model might achieve high accuracy simply by predicting the dominant class.

Precision: this metric is the number of correctly-identified members of a class divided by all the times the model predicted that class. In the case of *Ficus* dataset, the precision score is the number of correctly-identified cultivars divided by the total number of times the classifier predicted cultivars rightly or wrongly:

(7) $$\mathrm{Precision}=\frac{\mathrm{True}\;\mathrm{Positives}}{\mathrm{True}\;\mathrm{Positives}+\mathrm{False}\;\mathrm{Positives}}$$

Recall: is the number of members of a class that the classifier identified correctly divided by the total number of members in that class. For *Ficus* dataset, this would be the number of actual cultivars that the classifier correctly identified as such:

(8) $$\mathrm{Recall}=\frac{\mathrm{True}\;\mathrm{Positives}}{\mathrm{True}\;\mathrm{Positives}+\mathrm{False}\;\mathrm{Negatives}}$$

F1 score: combines precision and recall into one metric. If precision and recall are both high, F1 will be high, too. If they are both low, F1 will be low. If one is high and the other low, F1 will be low. F1 is a quick way to tell whether the classifier is actually good at identifying members of a class, or if it is finding shortcuts (e.g., just identifying everything as a member of a large class).

(9) $$F1\text{-}\mathrm{score}=2\cdot \frac{\mathrm{Precision}\cdot \mathrm{Recall}}{\mathrm{Precision}+\mathrm{Recall}}$$

A high F1-score indicates that the model is performing well in balancing precision and recall and is not overly biased toward one or the other.

#### 3.4.4. Software Used and Coding

Special procedures in Python language were also developed for the implementation of the data analyst which allowed us to derive general statistics, trends, and measure of the statistical significance, and to build the Random Forest framework. The implementation of the RF classifier was developed using class “RandomForestClassifier” of Scikit-Learn [50,51], adopting following hyperparameter: n_estimators = 100, criterion = ‘gini’, max_depth = None, min_samples_split = 2, min_samples_leaf = 1, min_weight_fraction_leaf = 0.0, max_features = ‘sqrt’, max_leaf_nodes = None, min_impurity_decrease = 0.0, bootstrap = True, oob_score = False, n_jobs = None, random_state = None, verbose = 0, warm_start = False, class_weight = None, ccp_alpha = 0.0, max_samples = None, monotonic_cst = None.

For the realization of the hierarchical clustering dendrograms and the bootstrap analysis, a linkage among cultivars was performed and this was followed by the transformation of the linkages into a tree structure. The linkage and the tree structure were derived using the ‘linkage’ and “to_tree” functions of Scipy-hierarchy [52], setting following parameters: method di linkage: “ward”; distance = “euclidean”; match_mode = “jaccard”, jaccard_threshold = “0.8”. Subsequently, 100 bootstrap replications were performed, recalculating the clustering each time, and finally a bootstrap support (in percentage) was derived for each node of the main dendrogram.

For data statistical analysis and production of tables and plots we used dedicated scientific python libraries, as such Pandas [53,54], Scipy [52], Statsmodels [55], and Penguin [56]. Plots were derived using Seaborn [57] and MatplotLib [58] Python modules.

## 4. Conclusions

The interesting aspect of the present study is the morphological analysis conducted on plants grown in a single environment; in fact, keeping plants in the same agro-environmental conditions for several years provides an important opportunity to describe phenotypic variation in accessions living in common conditions. In addition, it allows the study of phenotypic plasticity of long-lived species such as fig, in response to changing climatic conditions.

Leaf micromorphological characteristics, including the shape, size, composition, and density of trichomes on the epidermis, exhibit considerable variability. These features play a pivotal role in plant taxonomy and are highly discriminatory phenotypic traits for cultivar characterization, particularly when combined with other qualitative and quantitative morphological attributes [22,59].

The classification of fig (*Ficus carica*) cultivars is inherently complex due to the large number of traits to analyze—192 in total, comprising 126 qualitative and 66 quantitative variables—and the species’ unique characteristics, such as the presence of multiple leaf types [60]. Consequently, identifying a subset of reliable, discriminative variables represents a promising strategy for efficient fig germplasm characterization and classification. Nevertheless, focusing exclusively on leaf morphology still involves the analysis of numerous variables; 32 quantitative morphological variables were identified and evaluated.

To address this complexity, a machine learning-based approach employing the RF algorithm in conjunction with PCA was introduced. This methodology enabled the identification of the most effective variables for cultivar discrimination. Specifically, the study identified 10 variables per category, of which six were common across all 15 analyzed cultivars.

Among the identified traits, BAC, PLØ, and I2/TP exhibited the highest discriminative power, underscoring their potential importance in defining key structural differences between cultivars, likely associated with attributes such as lobe size, shape, or symmetry.

Preliminary findings of the research demonstrated the utility of machine learning for cultivar discrimination and suggested that automating the acquisition of morphological parameters, such as through a visual machine learning-based system, could further improve classification accuracy. To the best of our knowledge, this study represents the first application of RF combined with PCA in fig cultivar classification, and the results encourage further exploration of this approach for cultivars of other species.

## Figures and Tables

**Table 1:** Studied fig cultivars, abbreviations (code), qualitative descriptors included in morphological analysis.

Cultivar	Code	Leaf Margin	Shape of Central Lobe	Shape of Leaf Bas	Little Lobe in Central Lobe (%)	Little Lobe in Lateral Lobe (%)	N. of Lobes
ALBO	AL	crenate	lyrate- lanceolate	calcarate-cordate	11.11	44.44	5
BIANCO DI CARMIGNANO	BC	crenate	lanceolate	truncate -cordate	31.58	25.00	3 *
BROGIOTTO BIANCO	BB	crenate/dentato	lanceolate	cordate- calcarate	25.00	20.00	3 *
BROGIOTTO NERO	BN	crenate	lanceolate- romboidale	cordate calcarate-truncate	15.00	5.00	3
CORBO	CO	crenate	lanceolate- lyrate	calcarate	68.42	55.00	5
DOTTATO	DO	crenate	lanceolate	cordate	65.00	5.00	3 *
FIORONE	FI	crenate	lanceolate	cordate	18.18	100.00	3
GIGANTE DI CARMIGNANO	GI	crenate	lanceolate	cordate-truncate	65.00	31.58	3 *
PARADISO	PA	crenate	lanceolate	calcarate	20.83	8.70	3
PECCIOLOBIANCO	PB	undulate/crenate	lanceolate	cordate-calcarate	30.00	25.00	3 *
PECCIOLO NERO	PN	crenate	linear	cordate-calcarate	65.00	50.00	5
PERTICONE	PE	crenate	lanceolate- spatulate	cordate	45.45	59.09	5
PORTOGALLO	PO	crenate	lanceolate	truncate	82.35	78.57	3
SAN PIERO	SP	crenate	lanceolate	decurrente	45.00	15.00	3
VERDINO	VE	crenate	lanceolate	truncate	22.22	33.33	3

* Predominant type founded in the analyzed cultivars.

**Table 2:** Main statistic parameters of descriptor, sorted by CV. Explanation of leaf descriptors is given in Sez. 3.2-Morphological Descriptors.

Descriptor	N_o_ Sample	Mean	Standard Error	Standard Deviation	Median	Min	Max	1st Quartile	3th Quartile	CV
L3y (cm)	134	2.7	0.2	1.9	2.5	−3.8	9.8	1.5	3.8	72.5%
BAC (°)	287	117.8	3.1	52.7	110.0	15.2	270.0	85.0	157.5	44.6%
I3y (cm)	134	3.3	0.1	1.2	3.3	0.5	6.5	2.4	4.0	36.6%
WxH (cm^2^)	287	483.6	9.8	165.2	460.0	185.0	1156.0	371.9	567.8	34.1%
I2x (cm)	286	3.3	0.1	1.0	3.2	1.5	6.8	2.5	4.0	31.5%
I3x (cm)	134	6.5	0.1	1.6	6.4	2.6	10.5	5.5	7.5	24.2%
L3 (cm)	134	10.5	0.2	2.5	10.4	5.5	17.7	8.5	12.5	23.8%
I2_TP (cm)	287	9.8	0.1	2.3	9.8	4.4	16.0	8.2	11.5	23.5%
I2y (cm)	286	9.2	0.1	2.2	9.2	4.0	15.4	7.5	11.0	23.5%
L3x (cm)	134	10.0	0.2	2.3	10.0	5.2	15.1	8.0	11.8	23.0%
I2/L2	287	0.6	0.0	0.1	0.6	0.3	0.9	0.5	0.7	22.4%
I3_TP (cm)	134	7.4	0.1	1.6	7.4	3.5	11.3	6.2	8.8	22.1%
CLL (cm)	287	12.0	0.2	2.6	12.0	6.0	19.3	10.0	14.0	21.8%
Pl Ø (cm)	287	0.6	0.0	0.1	0.5	0.3	1.0	0.5	0.6	21.3%
PL (cm)	287	8.8	0.1	1.8	8.5	5.3	14.0	7.5	10.0	21.0%
PL/H	287	0.4	0.0	0.1	0.4	0.2	0.6	0.3	0.4	19.1%
L2y (cm)	287	13.5	0.2	2.6	13.3	7.0	20.8	11.7	15.2	19.0%
L2x (cm)	287	9.1	0.1	1.6	9.0	5.5	13.5	8.0	10.0	18.1%
PL/L1	287	0.4	0.0	0.1	0.4	0.3	0.7	0.4	0.5	18.1%
W (cm)	287	20.3	0.2	3.6	20.0	12.5	33.3	17.9	22.5	17.8%
α (°)	287	39.8	0.4	7.1	40.0	20.0	62.1	35.0	45.0	17.7%
L3/L1	134	0.5	0.0	0.1	0.5	0.3	0.7	0.4	0.5	17.3%
Zx (cm)	287	4.7	0.0	0.8	4.6	2.5	7.0	4.0	5.0	17.3%
I3/L3	134	0.7	0.0	0.1	0.7	0.5	1.0	0.6	0.8	17.3%
H (cm)	287	23.3	0.2	3.9	23.0	15.0	37.0	20.5	25.6	16.9%
L2_TP (cm)	287	16.4	0.2	2.7	16.3	9.7	24.1	14.4	18.2	16.6%
R (cm)	136	0.6	0.0	0.1	0.6	0.4	0.8	0.5	0.7	16.5%
Zy (cm)	287	14.1	0.1	2.3	13.9	7.7	20.0	12.5	15.6	16.3%
CLL/H	276	0.5	0.0	0.1	0.5	0.3	0.8	0.5	0.6	16.2%
Z_TP (cm)	287	14.9	0.1	2.3	14.6	9.3	20.8	13.2	16.5	15.4%
L1 (cm)	287	21.3	0.2	3.0	21.3	14.2	29.0	19.2	23.1	14.1%
β (°)	133	42.3	0.5	5.3	42.0	30.0	57.6	40.0	45.0	12.5%
L2/L1	287	0.8	0.0	0.1	0.8	0.5	1.0	0.7	0.8	8.4%

**Table 3:** Leaf descriptors of three-lobed cultivars. Codes for cvs are reported in Table 1; explanation of leaf descriptors is given in Sez. 3.2–Morphological Descriptors.

Descriptor	BC	BB	BN	DO	FI	GI	PA	PO	PB	SP	VE
PL (cm)	7.44 ± 1.2 b	9.57 ± 1.7 a	9.54 ± 1.9 a	8.52 ± 1.5 a–c	6.95 ± 1.5 c	8.74 ± 1.0 a–c	9.49 ± 1.5 a	8.52 ± 1.9 a–c	8.02 ± 1.4 a–c	8.62 ± 1.4 a–c	8.93 ± 1.8 ab
PL Ø (cm)	0.55 ± 0.1 ns	0.51 ± 0.06 ns	0.53 ± 0.07 ns	0.64 ± 0.1 ns	0.49 ± 0.1 ns	0.54 ± 0.09 ns	0.60 ± 0.1 ns	0.50 ± 0.1 ns	0.70 ± 0.08 ns	0.51 ± 0.09 ns	0.47 ± 0.08 ns
L1 (cm)	19.6 ± 2.5 c–e	24.0 ± 2.2 a	19.8 ± 2.8 c–e	22.8 ± 1.8 ab	18.4 ± 1.9 e	21.8 ± 2.7 a–d	19.6 ± 2.3 de	20.7 ± 1.8 b–e	21.6 ± 2.9 a–d	22.1 ± 2.3 a–c	20.1 ± 2.0 c–e
I2 x (cm)	2.83 ± 0.8 c	2.89 ± 0.4 c	4.39 ± 1.1 a	3.88 ± 0.6 ab	3.25 ± 0.8 bc	3.42 ± 1.1 bc	3.31 ± 0.7 bc	3.27 ± 0.4 bc	3.26 ± 1.2 bc	3.98 ± 1.1 ab	4.05 ± 0.9 ab
I2 y (cm)	7.76 ± 1.1 f	8.94 ± 1.2 d–f	9.38 ± 2.1 c–e	10.9 ± 1.3 ab	9.37 ± 1.5 c–f	9.75 ± 2.3 b–d	8.82 ± 1.6 d–f	11.3 ± 1.1 ab	7.91 ± 1.3 ef	11.4 ± 1.3 a	10.9 ± 1.0 a–c
I2_TP (cm)	8.30 ± 1.1 d	9.40 ± 1.2 cd	10.4 ± 2.1 bc	11.6 ± 1.3 ab	9.93 ± 1.6 b–d	10.4 ± 2.4 bc	9.43 ± 1.7 cd	11.8 ± 1.1 ab	8.61 ± 1.5 d	12.1 ± 1.5 a	11.6 ± 1.5 ab
L2 x (cm)	8.69 ± 1.4 b–d	10.5 ± 1.2 a	9.26 ± 1.2 a–d	9.94 ± 1.2 ab	7.52 ± 0.9 d	9.39 ± 1.7 a–c	8.21 ± 1.1 cd	7.89 ± 1.3 cd	10.5 ± 1.4 a	8.65 ± 1.6 b–d	8.04 ± 1.2 cd
L2 y (cm)	11.6 ± 2.2 c	15.0 ± 2.0 ab	12.3 ± 2.0 c	15.9 ± 1.8 a	11.6 ± 1.7 c	12.3 ± 2.2 c	12.3 ± 2.0 c	13.7 ± 1.8 a–c	12.7 ± 2.6 bc	13.4 ± 1.7 bc	13.0 ± 1.1 bc
L2_TP (cm)	14.5 ± 2.3 c	18.3 ± 2.1 ab	15.5 ± 2.2 c	18.8 ± 1.9 a	13.8 ± 1.8 c	15.7 ± 2.9 c	14.8 ± 2.2 c	15.9 ± 1.9 bc	16.6 ± 3.0 a–c	15.9 ± 2.1 bc	15.3 ± 1.8 c
Z x (cm)	4.27 ± 0.6 b	5.11 ± 0.7 a	5.09 ± 0.6 a	4.31 ± 0.5 b	5.05 ± 0.6 ab	4.69 ± 0.9 ab	4.37 ± 0.7 b	4.48 ± 0.7 ab	4.89 ± 0.6 ab	4.88 ± 0.4 ab	4.73 ± 0.7 ab
Zy (cm)	14.2.3 ± 2.4 ab	13.5 ± 1.6 ab	14.5 ± 2.3 ab	13.4 ± 2.0 ab	15.7 ± 1.8 a	13.4 ± 2.4 ab	13.0 ± 2.44 b	13.0 ± 1.2 ab	12.5 ± 2.7 b	14.2 ± 0.8 ab	11.1 ± 1.9 b
Z_TP (cm)	14.4 ± 3.2 a–c	14.5 ± 1.6	15.4 ± 2.1 ab	14.1 ± 1.9 a–c	16.5 ± 1.7 a	14.2 ± 2.6 a–c	13.7 ± 1.7 bc	14.4 ± 1.2 a–c	13.0 ± 3.2 c	15.0 ± 2.1 a–c	13.9 ± 1.8 a–c
CLL (cm)	11.8 ± 2.1 c	15.1 ± 1.3 a	10.4 ± 2.2 cd	11.8 ± 1.7 c	8.99 ± 1.1 d	12.1 ± 1.9 bc	10.8 ± 1.4 cd	9.46 ± 1.4 d	13.7 ± 2.6 ab	10.8 ± 1.6 cd	9.22 ± 1.8 d
W (cm)	18.3 ± 1.9 cd	22.7 ± 3.4 a	19.3 ± 2.6 b–d	21.5 ± 3.2 a–c	16.9 ± 2.5 d	21.7 ± 3.4 ab	18.6 ± 2.5 cd	18.3 ± 2.1 d	21.6 ± 3.2 a–c	19.3 ± 3.0 b–d	17.8 ± 3.3 d
H (cm)	21.2 ± 2.7 bc	25.5 ± 3.5 a	22.0 ± 2.9 bc	25.6 ± 3.0 a	19.7 ± 3.1 c	23.7 ± 3.0 ab	21.7 ± 2.8 bc	21.1 ± 2.0 bc	23.9 ± 4.0 ab	22.6 ± 2.4 a–c	21.4 ± 2.6 bc
BAC (°)	140.5 ± 45 c	113.6 ± 36 ef	100.0 ± 37 cd	98.7 ± 36 d	110.4 ± 42 ef	120.2 ± 37 cd	91.4 ± 18 d	183.3 ± 17 b	103.1 ± 25 d	226.1 ± 32 a	145.8 ± 39 bc
α (°)	40.1 ± 4.1 bc	41.3 ± 4.2 bc	41.9 ± 6.7 b	36.6 ± 4.3 bc	38.4 ± 6.2 b–d	42.5 ± 7.5 b	39.6 ± 4.7 bc	31.4 ± 3.1 de	49.4 ± 6.3 a	29.0 ± 6.4 e	36.0 ± 5.2 cd
CLL/H	0.56 ± 0.06 ab	0.60 ± 0.06 a	0.47 ± 0.07 cd	0.46 ± 0.05 cd	0.46 ± 0.05 cd	0.51 ± 0.07 bc	0.50 ± 0.05 bc	0.45 ± 0.04 cd	0.57 ± 0.07 a	0.48 ± 0.04 cd	0.43 ± 0.07 d
PL/H	0.35 ± 0.05 bc	0.38 ± 0.08 a	0.44 ± 0.09 a	0.32 ± 0.04 c	0.35 ± 0.03 a–c	0.37 ± 0.07 a–c	0.44 ± 0.07 a	0.40 ± 0.08 a–c	0.35 ± 0.1 bc	0.38 ± 0.04 a–c	0.42 ± 0.09 ab
WxH (cm^2^)	396.2 ± 82 de	580.6 ± 122 a	433.9 ± 95 c–e	558.9 ± 124 ab	338.3 ± 91 e	522.3 ± 141 a–d	410.5 ± 103 c–e	390.8 ± 103 c–e	527.5 ± 147 a–c	442.4 ± 112 b–e	388.5 ± 86 e
PL/L1	0.38 ± 0.07 b	0.39 ± 0.07 b	0.48 ± 0.1 a	0.37 ± 0.05 b	0.37 ± 0.03 b	0.40 ± 0.08 b	0.48 ± 0.07 a	0.41 ± 0.09 ab	0.38 ± 0.1 b	0.39 ± 0.05 b	0.44 ± 0.09 ab
L2/L1	0.74 ± 0.06 b	0.76 ± 0.04 ab	0.78 ± 0.08 ab	0.82 ± 0.04 a	0.75 ± 0.04 ab	0.72 ± 0.08 b	0.76 ± 0.09 ab	0.76 ± 0.06 ab	0.77 ± 0.09 ab	0.72 ± 0.05 b	0.76 ± 0.05 ab
I2/L2	0.58 ± 0.1 d–f	0.51 ± 0.04 f	0.67 ± 0.1 a–d	0.62 ± 0.06 c–e	0.72 ± 0.08 a–c	0.66 ± 0.1 a–d	0.64 ± 0.1 b–d	0.74 ± 0.08 ab	0.52 ± 0.08 ef	0.76 ± 0.08 a	0.77 ± 0.1 a

Data are the means ± SD (n = 20). In each column different letters represent significant differences (*p* < 0.05) according to Tukey’s HSD-test.

**Table 4:** Leaf descriptors of five-lobed cultivars. Codes for cvs are reported in Table 1; explanation of leaf descriptors is given in Sez. 3.2-Morphological Descriptors.

Descriptor	AL	CO	PN	PE
PL (cm)	9.07 ± 1.7 b	11.6 ± 1.6 a	8.01 ± 1.3 bc	7.58 ± 1.3 c
PL Ø (cm)	0.70 ± 0.09 a	0.72 ± 0.13 a	0.46 ± 0.07 b	0.47 ± 0.09 b
L1 (cm)	23.1 ± 2.4 a	24.9 ± 2.6 a	18.9 ± 2.6 b	20.3 ± 2.8 b
I2x (cm)	3.29 ± 1.5 a	3.40 ± 0.9 a	2.21 ± 0.5 b	2.41 ± 0.7 b
I2y (cm)	8.68 ± 1.9 ab	10.5 ± 2.9 a	5.91 ± 0.64 c	7.82 ± 2.1 b
I2_TP (cm)	9.32 ± 1.2 ab	12.0 ± 3.0 a	6.32 ± 0.7 c	8.21 ± 2.1 b
L2x (cm)	9.34 ± 1.89 ab	10.7 ± 1.7 a	8.43 ± 1.3 b	8.56 ± 1.4 b
L2y (cm)	14.3 ± 3.7 ab	16.4 ± 2.5 a	12.9 ± 2.8 b	13.9 ± 2.7 b
L2 _TP (cm)	17.5 ± 2.8 b	19.6 ± 2.5 a	15.4 ± 2.1 b	16.4 ± 2.9 b
I3x (cm)	7.31 ± 1.6 b	8.01 ± 1.0 a	4.89 ± 0.9 d	5.95 ± 1.2 c
I3y (cm)	3.21 ± 1.8 b	3.60 ± 0.6 ab	2.88 ± 0.7 b	4.28 ± 1.2 a
I3_TP (cm)	8.18 ± 1.6 ab	8.86 ± 0.9 a	5.71 ± 1.0 c	7.37 ± 1.5 b
L3x (cm)	10.9 ± 2.4 ab	12.2 ± 1.7 a	9.31 ± 1.5 b	10.9 ± 2.2 ab
L3y (cm)	2.76 ± 3.9 ab	2.95 ± 1.3 ab	2.88 ± 1.0 ab	4.12 ± 1.6 a
L3_TP (cm)	11.4 ± 2.2 ab	12.6 ± 1.8 a	9.9 ± 1.6 b	11.7 ± 2.4 ab
Zx (cm)	4.79 ± 1.4 a	5.17 ± 0.8 a	3.81 ± 0.7 b	4.46 ± 0.5 ab
Zy (cm)	15.1 ± 2.7 ab	16.3 ± 2.3 a	13.7 ± 2.0 b	14.0 ± 1.9 b
Z_TP (cm)	16.1 ± 2.5 ab	17.1 ± 2.2 a	14.2 ± 1.9 b	14.9 ± 1.9 b
CLL (cm)	14.3 ± 2.4 ab	14.4 ± 2.4 a	12.9 ± 2.3 ab	12.5 ± 2.0 b
W (cm)	23.7 ± 3.2 ab	24.6 ± 4.4 a	18.5 ± 2.5 c	20.7 ± 3.7 bc
H (cm)	26.1 ± 2.7 b	30.5 ± 3.9 a	20.5 ± 2.7 d	22.4 ± 3.3 cd
BAC (°)	91.5 ± 13 b	34.5 ± 12.8 c	113.4 ± 37 a	114.8 ± 23 a
α (°)	44.1 ± 8.0 ab	44.6 ± 4.0 a	39.7 ± 5.3 bc	37.9 ± 4.5 c
β (°)	41.4 ± 4.0 bc	45.4 ± 2.3 a	42.0 ± 5.2 ab	37.5 ± 4.1 c
CLL/H	0.56 ± 0.08 b	0.48 ± 0.08 c	0.63 ± 0.05 a	0.56 ± 0.07 b
PL/H	0.35 ± 0.05 b	0.35 ± 0.07 b	0.39 ± 0.05 a	0.34 ± 0.04 b
WxH (cm^2^)	623.6 ± 136 a	760.2 ± 223 a	385.9 ± 116 b	478.9 ± 145 b
PL/L1	0.39 ± 0.06 b	0.50 ± 0.09 a	0.43 ± 0.05 ab	0.38 ± 0.04 b
L2/L1	0.75 ± 0.13 ns	0.79 ± 0.07 ns	0.82 ± 0.06 ns	0.81 ± 0.07 ns
L3/L1	0.50 ± 0.21 b	0.51 ± 0.23 b	0.52 ± 0.17 ab	0.58 ± 0.06 a
I2/L2	0.56 ± 0.17 a	0.56 ± 0.13 a	0.41 ± 0.04 b	0.50 ± 0.06 ab
I3/L3	0.72 ± 0.04 a	0.71 ± 0.09 a	0.59 ± 0.05 b	0.64 ± 0.06 ab
R	0.69 ± 0.19 a	0.62 ± 0.10 ab	0.48 ± 0.05 c	0.55 ± 0.06 bc

Data are the means ± SD (n = 20). In each column different letters represent significant differences (*p* < 0.05) according to Tukey’s HSD -test.

**Table 5:** Results of an inferential statistical analysis, summarizing the outcomes of comparisons between groups or variables. The columns include the following: T: t-Student test statistic; *p*-val: *p*-value, indicating the statistical significance of the result; CI95%: 95% confidence interval, representing the range within which the true difference is likely to lie; Effect Size: a measure of the magnitude of the observed effect or difference between groups; BF10: Bayes Factor, comparing the likelihood of the alternative hypothesis to the null hypothesis; Power: statistical power, representing the probability of detecting a true effect if it exists; Class: classification of the effect size (e.g., Huge, Very large, Very small). The Table is divided into two sections: High Effect Size: reports comparisons with the three most significant effects (large effect sizes and very low *p*-values); Low Effect Size: includes comparisons with the last three smallest or negligible effects (very low effect sizes and high *p*-values). Codes for cvs are reported in Table 1; explanation of leaf descriptors is given in Sez. 3.2-Morphological Descriptors.

			T	*p*-val	CI95%	Effect Size	BF10	Power	Class Choen
High Effect Size	BAC	CO||SP	−25.5	1.59 × 10^25^	[−209.54 −178.75]	8.07	9.91 × 10^21^	1	Huge
		CO||PO	−34.5	3.00 × 10^25^	[−158.07 −140.4]	11.90	1.83 × 10^23^	1	Huge
		PA||PO	−21.2	4.05 × 10^20^	[−101.3 −83.52]	6.80	3.01 × 10^18^	1	Huge
	I2_TP	PN||SP	−17.5	8.91 × 10^20^	[−6.9 −5.47]	5.53	2.72 × 10^16^	1	Huge
		DO||PN	16.4	8.14 × 10^19^	[4.64 5.95]	5.18	3.23 × 10^15^	1	Huge
		PN||PO	−17.0	3.63 × 10^13^	[−6.09 −4.76]	6.47	2.02 × 10^14^	1	Huge
	I2y	PN||SP	−19.6	1.95 × 10^21^	[−6.01 −4.88]	6.19	1.09 × 10^18^	1	Huge
		DO||PN	16.1	1.47 × 10^18^	[4.43 5.7]	5.09	1.82 × 10^15^	1	Huge
		PB||SP	−10.7	5.37 × 10^13^	[−4.26 −2.91]	3.38	8.62 × 10^9^	1	Huge
	PL	BC||CO	−9.0	5.33 × 10^11^	[−5.05 −3.2]	2.86	1.11 × 10^8^	1	Huge
		CO||FI	9.9	8.65 × 10^11^	[3.66 5.56]	3.22	6.88 × 10^7^	1	Huge
		CO||PE	8.7	1.93 × 10^10^	[3.06 4.91]	2.72	7.26 × 10^7^	1	Huge
	PL_L1	CO||PE	7.5	4.9 × 10^9^	[0.06 0.11]	2.34	2.16 × 10^6^	1	Huge
		PA||PE	7.1	1.95 × 10^8^	[0.07 0.13]	2.05	1.03 × 10^6^	1	Huge
		DO||PA	−6.2	2.13 × 10^7^	[−0.14 −0.07]	1.86	5.24 × 10^4^	1	Very large
	WxH	BC||CO	−6.9	3.0 × 10^8^	[−479.15 −262.56]	2.19	3.02 × 10^5^	1	Huge
		CO||PN	6.7	6.02 × 10^8^	[261.43 487.17]	2.12	1.61 × 10^5^	1	Huge
		CO||FI	7.2	7.28 × 10^8^	[301.84 542.02]	2.22	1.38 × 10^5^	1	Huge
Low Effect Size	BAC	BN||DO	0.1	0.92	[−21.5 23.82]	0.03	0.31	0.0512	Very small
		AL||PA	0.0	0.96	[−8.45 8.87]	0.01	0.306	0.0503	Very small
		BB||PN	0.0	1.00	[−22.4 22.35]	0.00	0.309	0.0500	Negligible
	I2_TP	BC||PE	0.2	0.81	[−0.96 1.21]	0.07	0.31	0.0558	Very small
		BB||PA	0.0	0.97	[−0.81 0.78]	0.01	0.299	0.0502	Very small
		DO||VE	0.0	0.99	[−0.92 0.94]	0.01	0.315	0.0500	Negligible
	I2y	BC||PB	0.1	0.93	[−0.63 0.69]	0.03	0.31	0.0509	Very small
		PB||PE	0.0	0.97	[−1.09 1.04]	0.01	0.304	0.0502	Very small
		BC||PE	0.0	0.99	[−1.05 1.07]	0.00	0.303	0.0500	Negligible
	PL	BB||BN	0.1	0.96	[−1.16 1.22]	0.02	0.309	0.0503	Very small
		PB||PN	0.0	0.99	[−0.86 0.87]	0.00	0.309	0.0500	Negligible
		DO||PO	0.0	1.00	[−1.29 1.29]	0.00	0.333	0.0500	Negligible
	PL_L1	BB||GI	−0.1	0.92	[−0.04 0.04]	0.03	0.31	0.0510	Very small
		FI||PE	0.1	0.94	[−0.02 0.03]	0.03	0.348	0.0505	Very small
		AL||SP	0.1	0.94	[−0.03 0.04]	0.02	0.316	0.0506	Very small
	WxH	PO||VE	0.1	0.94	[−57.68 62.31]	0.03	0.339	0.0507	Very small
		BC||PO	0.0	0.96	[−59.82 57.02]	0.02	0.333	0.0503	Very small
		BC||VE	0.0	0.97	[−55.73 57.56]	0.01	0.315	0.0501	Very small

**Table 6:** Classification Report. The codes of the cultivars are reported in Table 1.

Cultivars	Precision	Recall	F1-Score
AL	0.50	0.20	0.29
BC	0.40	0.40	0.40
BB	0.43	0.60	0.50
BN	0.00	0.00	0.00
CO	0.71	1.00	0.83
DO	0.25	0.40	0.31
FI	1.00	0.33	0.50
GI	0.00	0.00	0.00
PA	0.44	0.67	0.53
PB	0.75	0.60	0.67
PN	0.29	0.33	0.31
PE	0.60	0.60	0.60
PO	0.60	1.00	0.75
SP	1.00	0.80	0.89
VE	0.67	0.50	0.57
weighted average	0.49	0.49	0.47
accuracy	0.49		

**Table 7:** The first three components from the Principal Component Analysis (PCA) of the six most significant characters of all fifteen cvs studied. Explanation of leaf descriptors is given in Sez. 3.2-Morphological Descriptors.

Trait	PC1	PC2	PC3
WxH	0.45	−0.12	−0.55
PL	0.48	−0.39	0.24
I2_TP	0.50	0.41	0.11
I2y	0.51	0.41	0.09
PL_L1	0.19	−0.51	0.60
BAC	−0.16	0.49	0.51
Eingen Values	2.55	1.8	1.14
% of variance	42.4	30.1	19
Cumulative variance (%)	42.4	72.5	91.5

**Table 8:** Trichome density (mm^2^) of upper and lower epidermis of leaves. The codes of the cultivars are reported in Table 1.

Trichomes Density (mm^2^)		
Cv	Upper Epidermis	Lower Epidermis
AL	26.3 ± 3.26 a	48.5 ± 2.38 f
BB	2.02 ± 0.77 ef	71.5 ± 2.65 b
BC	7.52 ± 1.5 b	64.7 ± 2.78 c
BN	3.50 ± 0.51 d–f	64.5 ± 2.88 c
CO	4.25 ± 035 c–f	32.2 ± 2.18 h
DO	7.48 ± 1.02 bc	63.2 ± 1.90 c
FI	5.02 ± 0.76 b–e	23.2 ± 2.91 i
GI	2.04 ± 0.89 ef	54.2 ± 2.65 ef
PA	3.26 ± 0.46 d–f	93.8 ± 1.79 a
PB	2.50 ± 0.35 d–f	77.0 ± 1.86 b
PE	5.53 ± 0.58 b–d	87.5 ± 1.10 a
PN	3.01 ± 0.36 d–f	55.8 ± 1.56 de
PO	3.02 ± 0.32 d–f	61.5 ± 2.41 cd
SP	2.76 ± 0.28 d–f	39.7 ± 0.68 g
VE	1.02 ± 0.12 f	48.2 ± 1.14 f

Data are the means ± SD. In each column different letters represent significant differences (*p* < 0.05) according to Tukey’s HSD-test.

**Table 9:** Abbreviation and units of morphometric parameters.

Abbreviation	Description	Units
H	Lamina length	cm
W	Lamina width	cm
WxH	Area: leaf length × width	cm^2^
PL	Petiole length	cm
PLØ	Petiole diameter	cm
CLL	Length of the central lobe	cm
BAC	Petiole sinus: angle between left and right basal lobe	°
α	Angle between L1 and L2	°
β	Angle between L2 and L3;	°
Z_TP	Central lobe maximum width calculated using the Pythagorean Theorem applied to Zx; Zy	cm
Zx	x coordinate of the point Z on the Cartesian plane	cm
Zy	y coordinate of the point Z on the Cartesian plane	cm
L1	Apex of the central lobe, coincides with L1y	cm
L2_TP	Apex of the secondary lobe calculated using the Pythagorean Theorem applied to Lx and Ly	cm
L2x	x coordinate of the point L2 on the Cartesian plane	cm
L2y	y coordinate of the point L2 on the Cartesian plane	cm
I2_TP	Sinus 2 calculated using the Pythagorean Theorem applied to I2x; I2y	cm
I2x	x coordinate of the point I2 on the Cartesian plane	cm
I2y	y coordinate of the point I2 on the Cartesian plane	cm
L3_TP	Apex of the tertiary lobe calculated using the Pythagorean Theorem applied to L3x; L3y	cm
L3x	x coordinate of the point L3 on the Cartesian plane	cm
L3y	y coordinate of the point L3 on the Cartesian plane	cm
I3_TP	Sinus 3 calculated using the Pythagorean Theorem applied to I3x; I3Y	cm
I3x	x coordinate of the point I3 on the Cartesian plane	cm
I3y	y coordinate of the point I3 on the Cartesian plane	cm
I2/L2	Ratio between sinus 2 (I2_TP) and apex of secondary lobe (L2_TP)	
L2/L1	Ratio between apex of secondary lobe (L2_TP) and apex of central lobe (L1)	
I3/L3	Ratio between sinus 3 (I3_TP) and apex of tertiary lobe (L3_TP)	
R	(I2_TP + I3_TP)/(L2_TP + L3_TP)	
PL/H	Ratio between petiole length and lamina length	
PL/L1	Ratio between petiole length and apex of central lobe	
CLL/H	Ratio between central lobe length and lamina length	

## Data Availability

All data will be provided upon request to the authors.

## Supplementary Materials

The following supporting information can be downloaded at: https://www.mdpi.com/article/10.3390/plants14030333/s1, Table S1: Percentage length distribution of non-glandular trichomes in the lower and upper leaf epidermis of 15 cultivars of *Ficus carica*.
