# Supplementary material for: Description of Ficus carica L. Italian Cultivars—I: Machine Learning Based Analysis of Leaf Morphological Traits
Source: Plants (Basel). 2025 Jan 23;14(3):333. doi: 10.3390/plants14030333 (PMC11819687; doi:10.3390/plants14030333)
Supplement: Supplementary file 1 [file plants-14-00333-s001.zip › plants-3379101-supplementary.pdf]

Supplementary Materials

Table S1 Percentage length distribution of non-glandular trichomes in the lower and upper leaf epidermis of 15 cultivars of *Ficus carica*. Trichome lengths were distributed into 11 width classes: class no. 1: 0.1-99 µm; class no. 2: 100-140 µm; class no. 3: 140.1-159 µm; class no. 4: 160-239 µm; class no. 5: 240-299 µm; class no. 6: 300-319 µm; class no. 7: 320-332 µm; class no. 8: 334-358 µm; class no. 9: 360-398 µm; class no. 10: 412-438 µm; class no. 11: 450-477 µm. Codes for cultivars are reported in ***Error! Reference source not found.***.

	CLASS										
**Cultivar**	**1**	**2**	**3**	**4**	**5**	**6**	**7**	**8**	**9**	**10**	**11**
**UPPER EPIDERMIS**											
AL	40,95	19,05	3,81	29,52	5,71						0,95
BC	50,00	30,00	6,67	13,33							
BB	50,00	12,50	12,50	12,50		12,50					
BN	42,86	35,71		21,43							
CO	94,12	5,88									
DO	13,33	20,00	13,33	43,33	10,00						
FI	95,00		5,00								
GI	37,50	25,00	25,00		12,50						
PA	76,92	15,38			7,69						
PB	40,00	20,00	20,00	10,00	10,00						
PN	33,33	33,33	8,33	25,00							
PE	36,36	31,82	4,55	27,27							
PO	16,67	33,33	16,67	33,33							
SP	100,00										
VE	50,00	25,00		25,00							
**LOWER EPIDERMIS**											
AL	19,59	26,80	9,28	30,41	9,79	2,06	0,52	1,03	0,52		
BC	12,74	11,20	11,58	45,17	15,44	3,09	0,39	0,39			
BB	24,20	31,32	14,59	25,62	4,27						
BN	45,35	24,81	8,14	18,99	2,71						
CO	25,58	27,91	11,63	33,33	1,55						
DO	22,22	19,05	9,13	29,37	13,49	2,78	1,19	1,98	0,40	0,00	0,40
FI	76,34	20,43	3,23								
GI	19,35	25,81	11,52	28,11	6,45	1,84	2,76	1,84	1,84	0,46	
PA	28,27	21,07	8,27	32,53	7,47	0,80	1,07	0,27	0,27		
PB	31,17	29,87	10,71	23,38	4,22	0,65					
PN	19,73	21,97	13,45	34,98	5,83	1,35		0,90	1,35	0,45	
PE	20,86	25,14	13,43	30,29	10,00	0,29					
PO	12,20	14,63	8,13	26,42	25,61	4,07	1,22	2,85	2,03	1,63	1,22
SP	64,15	25,79	3,77	5,03	1,26						
VE	87,50	11,46	0,52	0,52							
